# Plastic Responses to Single and Combined Environmental Stresses in a Highly Chemodiverse Aromatic Plant Species

**DOI:** 10.1111/ppl.70626

**Published:** 2025-11-05

**Authors:** Xue Xiao, Thomas Dussarrat, Dominik Ziaja, Yonca B. Seymen, Lukas Brokate, Ruth Jakobs, Baris Weber, Jana Barbro Winkler, Jörg‐Peter Schnitzler, Caroline Müller

**Affiliations:** ^1^ Bielefeld University Bielefeld Germany; ^2^ Research Unit Environmental Simulation Helmholtz Zentrum Munich Neuherberg Germany; ^3^ Joint Institute for Individualisation in a Changing Environment (JICE) University of Münster and Bielefeld University Bielefeld Germany

**Keywords:** Asteraceae, chemodiversity, combined stresses, essential oil, intraspecific variation, phenotypic plasticity

## Abstract

Plants face various environmental stresses, to which they respond in different ways. Due to climate change, it is expected that plants will encounter increased phases of drought and changes in herbivory. This study thus aimed to evaluate the intra‐individual variation in responses, that is phenotypic plasticity, to single and combined stresses, including drought and insect herbivory. We used plants of the aromatic species *Tanacetum vulgare* , which are characterized by distinct terpenoid chemotypes and metabolic fingerprints shaped by maternal origin. Clones were exposed to no stress, drought, herbivory, or a combination of both. The impacts of these treatments were determined in terms of aboveground biomass as well as emission rates or concentrations, richness, and functional Hill diversity (FHD) of volatile organic compounds (VOCs), stored leaf and root terpenoids, and leaf metabolic fingerprints. Drought resulted in lower plant aboveground biomass, VOC richness, and VOC FHD. Herbivory had no effect on biomass, but increased the VOC emission rates and richness, also in combination with drought. The treatment significantly affected the phenotypic plasticity of the aboveground biomass and VOC emission. Our findings highlight the importance of studying intra‐individual variation in plant responses to different stresses and their combinations to fully comprehend the finely tuned chemodiversity.

## 1 Introduction

Unlike animals, plants are immobile and therefore need to respond rapidly and dynamically to changing environments (Karban, Grof‐Tisza, and Blande 2016). Under the ongoing global climate change, plants face, for example, prolonged periods of drought in many regions worldwide (Ionita and Nagavciuc 2021; IPCC 2022). Phenotypic plasticity is crucial for plants, enabling them to cope with different environmental stressors imposed by global climate change through adjusting their growth and metabolism. For instance, in response to drought, plants often show retarded shoot growth (Chaves et al. 2003; Osakabe et al. 2014). In response to drought but also to herbivory, plants modulate various specialized (also called secondary) metabolites (Kaplan et al. 2008; Ahmad et al. 2023), including changes in the emission of volatile organic compounds (VOCs) (Arimura et al. 2009; Reinecke et al. 2024). So far, most studies have focused on plant responses to individual rather than combined stresses (Kleine and Müller 2014; Scott et al. 2019). Even fewer studies accounted for phenotypic plasticity in the strict sense, that is the capacity of a given genotype to render different phenotypes (Valladares et al. 2006), in such responses. This underlines the importance of controlled experiments testing variation in stress responsiveness by using a wide range of ecotypes or genotypes to draw conclusions about intraspecific variation in phenotypic plasticity.

Within some plant species, an intriguing diversity in metabolites can be found among individuals. This diversity, known as chemodiversity, is determined by the number of metabolites (metabolic richness), their abundance and their specific composition (Müller and Junker 2022). While the inducibility of individual phytochemicals has been well investigated in recent decades (Schaller 2008; Calf et al. 2020), the influence of abiotic and biotic stresses on different chemodiversity indices has received less attention. In response to drought, both increases and decreases were found in Shannon diversity and richness of VOCs in various grassland species compared to well‐watered individuals, with highly species‐specific responses (Reinecke et al. 2024). Co‐occurring drought and herbivory can also interactively affect plant chemical composition (Kleine and Müller 2014; Nguyen et al. 2016; Gely et al. 2020; Lin et al. 2022), which may affect chemodiversity. Thus, the specific phytochemical composition may play an important role in shaping an individual's niche and fundamentally influencing its fitness due to the pivotal functions the various metabolites have in interactions with the environment (Müller and Junker 2022).

Many VOCs are terpenoids, which are the largest class of specialized metabolites, being both structurally and functionally highly diverse (Zhou and Pichersky 2020). Next to being released as VOCs, terpenoids can be stored in glandular trichomes or other tissues (Clancy et al. 2016; Tissier et al. 2017). Drought has been shown to induce various responses in terpenoid concentrations across species, including increases, decreases, and no measurable response (Kleine and Müller 2014; Jud et al. 2016; Reinecke et al. 2024), suggesting species‐specific adaptations. Herbivory typically increases the release of VOCs that attract natural predators and “alert” neighboring plants (Kost and Heil 2006; Gols 2014). Fewer studies have investigated the induction of stored terpenoids. However, an increase in both terpenoid concentrations and the density of terpenoid‐storing glands has been found in cotton leaves in response to herbivory (Eisenring et al. 2017). Additionally, root terpenoid concentrations can be affected by aboveground herbivory and drought. For instance, in *Solanum lycocarpum* infested by *Spodoptera exigua* caterpillars, root terpenoid concentrations increased in well‐watered, but decreased in drought‐stressed plants (Mundim et al. 2021). These findings emphasize the finely tuned nature of plant chemical responses to individual and combined stresses.

*Tanacetum vulgare* (common tansy, Asteraceae) is a widespread aromatic plant species that exhibits a high intraspecific chemodiversity in leaf terpenoid profiles (Clancy et al. 2016; Ziaja and Müller 2023), forming distinct chemotypes (Keskitalo et al. 2001). The terpenoids are stored in glandular trichomes on the leaf surface but are also constitutively released as VOCs (Jakobs and Müller 2019). As an outcrossing species, offspring from one mother plant can belong to different chemotypes. These maternal origins also influence the chemical composition, apart from terpenoids, substantially (Dussarrat et al. 2023). Since *T. vulgare* can be propagated clonally, this species is well‐suited for testing phenotypic plasticity. Previous research revealed that *T. vulgare* plants from 18 populations showed overall no growth effects in response to drought under field conditions (Kleine and Müller 2014; Kleine et al. 2017). However, under controlled conditions, plants of two chemotypes exposed to drought for twelve days produced less aboveground biomass compared to well‐watered plants (Kleine and Müller 2014). Total leaf terpenoid concentrations decreased slightly in response to drought but were unaffected by herbivory from *Mamestra brassicae* larvae. Furthermore, the relative amounts of the main leaf terpenoids dominating the two chemotypes, β‐thujone, and *trans*‐carvyl acetate, respectively, used in the experiment were unaffected by drought and herbivory. In contrast, root terpenoid concentrations were significantly enhanced by both drought and herbivory (Kleine and Müller 2014). Despite these findings, a comprehensive approach testing responses in various metabolite classes across multiple chemotypes of different maternal origin remains lacking.

In the present study, we aimed to assess the capacity of *T. vulgare* to cope with single and combined stresses in a highly controlled setting. Therefore, we exposed clones of *T. vulgare* plants of four chemotypes from different maternal origins to one of the following four treatments: control, drought, insect herbivory, or drought plus insect herbivory (combined). We examined biomass and a suite of chemical traits, including VOC emissions, stored leaf, and root terpenoid profiles as well as leaf metabolic fingerprints, to evaluate how responses varied by treatment, chemotype, and maternal origin. We hypothesized that plant responses are treatment‐specific, that the combined treatment exerts a more pronounced response than individual stresses, and that the magnitude and direction of responses differ across chemotypes and maternal origins, reflecting high intraspecific plasticity.

## 2 Materials and Methods

### 2.1 Plant and Insect Rearing

For the experiment, *Tanacetum vulgare* plants of four chemotypes, originating from six distinct maternal origins (offspring of six different mother plants, Table S1), were chosen from a stock established at Bielefeld University (for further details see Ziaja and Müller 2023). Each maternal origin (numbers 7, 8, 16, 18, 23, 26) was represented by plants of two chemotypes, occurring in different combinations. Chemotypes had been classified based on their foliar terpenoid composition. These chemotypes were dominated (that is > 50% of total terpenoid composition belonged to one terpenoid = mono chemotype, or three terpenoids = mixed chemotype) by artemisia ketone (from here on referred to as “Keto” chemotype; mono chemotype), β‐thujone (“Bthu,” mono chemotype), artemisyl acetate, artemisia ketone and artemisia alcohol (“Aacet,” mixed chemotype) or (*Z*)‐myroxide, santolina triene, and artemisyl acetate (“Myrox,” mixed chemotype). In order to obtain a sufficient number of clones (up to 16) per plant individual, two rounds of root cuttings were performed (2 months apart; for timetable of experiment see Figure S1). Plants were grown in pots with a steamed 1:1 mixture of sand: soil (Fruhstorfer Einheitserde Typ T, Gebr. Patzer GmbH & Co. KG). Two weeks after the second set of root cuttings had been prepared, the plants were sent to Munich and potted there in a 4:4:1:1 mixture of sand: soil: vermiculite: perlite. The pot size was adjusted according to plant development, starting with 6 × 6 × 6 cm, via 9 × 9 × 9 cm during acclimatization, up to 13 × 13 × 13 cm pots used during the stress treatments. After acclimatization in the greenhouse for 1 month, plants were transferred into the four phytotron chambers of the ExpoSCREEN facility (Roy et al. 2021), each consisting of four sub‐chambers. The climatic conditions simulated average conditions in June, that is 25°C/14.5°C, 40%/80% relative humidity (r.h.) (day/night) and 16 h of daylight. The light increased during 3.5 h to approx. 718 μmol m^−2^ s^−1^ photosynthetic flux density, and decreased in the afternoon until nightfall (again 3.5 h) to simulate the natural light conditions from sunrise to sunset. The course of the day's climatic conditions was shifted by 1 h in each of the four chambers so that sampling could take place under the same conditions in each chamber.

Eggs of *Spodoptera exigua* were received from a long‐term laboratory rearing and larvae were kept on leaves of greenhouse‐grown, nonflowering, six‐week‐old Chinese cabbage ( *Brassica rapa* var. *pekinensis*) in ventilated boxes in a climate cabinet at 22°C, 16:8 L:D, 60% r.h. Fresh leaves were added every other day and larvae were provided with food *ad libitum*. Larvae for the experiment were used 3 weeks after hatching.

### 2.2 Experimental Set‐Up

Clonal plants of similar size were assigned to one of the four treatments: control, drought, insect herbivory, and drought plus herbivory (called combined treatment in the following), and each clone was placed in one sub‐chamber within one—up to four—of the main chambers (Figure S2). About 2 weeks after transferring plants into the sub‐chambers, plants were phenotyped by taking pictures from different angles in a photo‐station, a cubic housing with a blue background, two cameras and a lighting unit (as described in Jud et al. 2018). One camera was positioned at a 45° angle above the plant, while the other camera had a horizontal view of the plants. To determine the leaf area, the plants were placed on a turntable and seven images of each plant were taken from different angles (*i* * 51.43°) during a 360° rotation. This arrangement allowed for standardized lighting conditions during image acquisition and a complete picture of the plants.

After replacing the plants in the sub‐chambers, the drought treatment started. Plants of the drought and combined treatment were only watered with approximately half of the amount of water the control and herbivory‐treated plants received until the end of the experiment (Table S2). Two weeks later, all plants were phenotyped in the photo‐station again. Afterwards, a roasting bag (80 cm height, RUBIN; Dirk Rossmann GmBH) was placed around each plant, being open to the top and at least as high or higher than the plants (30 cm above the top rim of each pot) and plants were placed back into the sub‐chambers. Two days later, groups of three larvae of *S. exigua* (one larger, likely 4th instar, and two smaller, likely 3rd instar) were weighed and placed on all plants of the herbivory and combined treatment 1 h after the onset of light. For VOC collection 3 days after the start of the herbivory treatment, polydimethylsiloxane (PDMS)‐coated stir bar “twisters” (Gerstel GmbH) were fixed on sticks within the canopy of each plant. The roasting bags were slightly closed with one clip at the top. VOCs were collected under static conditions from each individual plant for 6 h without additional airflow (as in Clancy et al. 2016; Eckert et al. 2023), starting 5.5 h after the onset of light. VOCs collected in roasting bags fixed on empty pots served as background samples. Twisters were stored at +4°C until VOC analysis (see below). Five days after placing the larvae on the respective plants, the roasting bags were removed from all plants, larvae were removed and their survival was assessed. One day later, the plants were photographed again.

### 2.3 Harvest

Two days after herbivore removal, the second youngest leaf was harvested for subsequent terpenoid analysis and the third leaf for metabolic fingerprinting. Leaves were placed in aluminium foil and frozen in liquid nitrogen. The remaining aboveground biomass was harvested, weighed, dried at 70°C for 3 days and weighed again. To analyze root terpenoid profiles, the root system was cut at ground level, root sections were randomly taken, and soil was removed with a brush. These root samples were placed in aluminium foil and frozen in liquid nitrogen. Plant material for chemical analyses was lyophilized for 48 h and weighed. Leaf samples were weighed and biomass was added to the remaining aboveground dry biomass to determine the total aboveground biomass.

### 2.4 Volatile Analyses

VOCs of samples and blanks collected on the PDMS tubes were measured with a thermal‐desorption unit (TDU) combined with a cryo injection system (CIS) (Gerstel GmbH & Co. KG) coupled to a gas chromatograph with mass spectrometer (Agilent 7890A GC and 5975C MS, Agilent Technologies) equipped with a DB‐5 ms column (70 m × 250 μm, 0.25 μm‐film, 14% cyanopropyl‐phenyl‐methylpolysiloxane with 10 m guard column; Agilent Technologies). Prior to the analysis, the internal standard δ‐2‐carene was added to the twisters. Analytes were thermally desorbed from 30°C to 270°C (2 min hold) in the TDU, cryofocused in the CIS liner filled with Tenax and glasswool at −50°C, then released to 270°C at 12°C s^−1^ with a 2 min hold. Helium at 1 mL min^−1^ served as carrier gas. The oven program started at 40°C, ramped to 130°C at 10°C min^−1^ (5 min hold), then to 175°C at 80°C min^−1^, to 200°C at 2°C min^−1^, to 220°C at 4°C min^−1^, and finally to 300°C at 100°C min^−1^ (5 min hold). Data were processed with Agilent MSD ChemStation (E.02.01; Agilent Technologies). For tentative identification, we used the National Institute of Standards and Technology (NIST) 20 and Wiley 275 libraries and Kováts indices derived from a C7–C30 alkane mixture (only C8–C24 visible) measured at the same conditions (Sigma‐Aldrich). All VOCs, including terpenoids and unknown compounds, were normalized to the internal standard δ‐2‐carene, and the average of the mean peak areas of the blanks was subtracted from the peak areas of the plant samples. The resulting values (signal intensities) were normalized to the total leaf area of the corresponding plants and the sampling duration.

### 2.5 Stored Terpenoid Analyses

For liquid extraction of stored terpenoids, 10 ± 1 mg of leaves and 55 ± 5 mg of root material were taken, respectively, extracted and homogenized in 1 mL (leaves) or 0.5 mL (roots) of *n*‐heptane (Honeywell Deutschland) containing 10 ng μL^−1^ of 1‐bromodecane as an internal standard in a FastPrep‐24 5G (MP Biomedicals Germany) for 30 s at 9.00 m s^−1^. Root samples were milled prior to this extraction for 2 × 30 s at 30 Hz (Retsch MM 301). Samples were sonicated for 5 min (leaves) or 15 min (roots) in an ultrasonic bath (Emmi‐H30; emmi EMAG) at room temperature, and then centrifuged for 10 min at 16,110 rcf. Samples and blanks were injected with a split of 5 into a GC–MS (GC 2010plus—MS QP2020, Shimadzu, with VF‐5 MS column, 30 m × 0.2 mm inner diameter, with 10 m guard column, Varian), with electron impact ionization mode at 70 eV and helium as carrier gas with a flow rate of 1.5 mL min^−1^. The initial temperature of 50°C was held for 5 min, increased to 250°C at 10°C min^−1^, further to 280°C at 30°C min^−1^, and finally held for 3 min. Blanks containing only the extraction solvent were also measured. In 19 out of 33 samples of plants of the Keto chemotype, artemisia ketone peaks were overloaded. These samples were measured again with a split of 10 or 20, depending on the magnitude of overload.

Chromatograms were analyzed using LabSolutions GCMS Postrun Analysis v4.45 (Shimadzu). Compounds were annotated by comparing the retention indices (RI) and mass spectra with library entries of NIST (2014), the Flavour and Fragrance Natural and Synthetic Compounds GC/MS Library (FFNSC 3.0 Shimadzu), to values reported in Adams (2007), and to compounds previously annotated in clones of these plants (Ziaja and Müller 2023). Compounds not identified previously were named “unknown monoterpenoid” or “unknown sesquiterpenoid,” if their RI deviated > 1.5% from the RI reported in Adams (2007). Quantification of the compounds was based on the extracted ion chromatogram of the target ion. Terpenoids were normalized by the internal standard; the average of the peak areas in the blanks was subtracted from the peak areas of the plant samples, and compounds were normalized to the sample dry mass.

### 2.6 Leaf Metabolic Fingerprint Analyses

For analyses of leaf metabolic fingerprints by UHPLC‐QTOF‐MS/MS (UHPLC: Dionex UltiMate 3000, Thermo Fisher Scientific; QTOF: compact, Bruker Daltonics), dried leaf material was homogenized and 8 ± 2 mg extracted in methanol 90% (v:v) containing hydrocortisone as an internal standard (Sigma‐Aldrich) by sonicating in an ice bath for 15 min. Supernatants were collected, centrifuged for 10 min and filtered using 0.2 μm filters (Phenomenex).

Samples and blanks were analyzed on a Kinetex XB‐C18 column (150 × 2.1 mm, 1.7 μm, with guard column; Phenomenex) at 45°C and a flow rate of 0.5 mL min^−1^ using a gradient from eluent A [Millipore‐H_2_O with 0.1% formic acid (FA)] to eluent B (acetonitrile with 0.1% FA): 2 to 30% B within 20 min, increase to 75% B within 9 min, followed by column cleaning and equilibration, as described in Schweiger et al. (2021). The QTOF was operated in negative electrospray ionization mode at a spectra rate of 6 Hz in the *m*/*z* (mass‐to‐charge) range of 50–1300. The settings for the MS mode were: end plate offset 500 V, capillary voltage 3000 V, nebulizer (N_2_) pressure 3 bar, dry gas (N_2_; 275°C) flow 12 L min^−1^, low mass 90 *m*/*z*, quadrupole ion energy of 4 eV and a collision energy of 7 eV. The Auto MS/MS mode was used to obtain MS/MS spectra, ramping the isolation width and collision energy along with increasing *m*/*z*. Additional MS/MS analyses of some samples were performed to target selected ions (that is best markers) using multiple reaction monitoring (MRM). A calibration solution with sodium formate was used for the recalibration of the *m*/*z* axis. The processing of the LC–MS data was performed using the T‐ReX 3D algorithm of MetaboScape (v. 2021b, Bruker Daltonics). Settings included the presence of features in a minimum of 3 samples, a correlation coefficient threshold (ESI correlation) of 0.8, an intensity (peak height) threshold of 1000 and a minimum peak length of 11. Features were normalized by dividing the peak heights by the height of the hydrocortisone [M + HCOOH—H]^−^ ion, signals present in blank samples were subtracted, and feature intensities were divided by the sample dry mass. The feature file containing full MS and MS/MS data was uploaded to the Global Social Molecular Network of Natural Products (GNPS, https://gnps.ucsd.edu) platform for molecular networking using the online feature‐based molecular networking module (FBMN). Features were connected by a similarity score of their MS/MS spectra (the parent ion and fragment ion error were 0.02 Da, the smallest matching cosine value was 0.7). A total of 407 features were included in the network analysis, and the resulting similarity matrix was converted into a dissimilarity matrix to calculate the functional Hill diversity (FHD) (Petrén et al. 2023).

### 2.7 Statistical Analyses

All statistical analyses were conducted in R v4.5.0 (R Developmental Core Team, 2025), using the packages multcomp (Hothorn et al. 2008), multcompView (Graves et al. 2024), vegan (Oksanen et al. 2025), dplyr (Wickham et al. 2023), lmPerm (Wheeler and Torchiano 2025), nlme (Pinheiro and Bates 2025), car (Fox and Weisberg 2019), chemodiv (Petrén et al. 2023), MuMin (Bartoń 2025), ropls (Thévenot et al. 2015), and rstatix (Kassambara 2023). Visualization was done with ggplot2 (Wickham 2016) and patchwork (Pedersen 2024).

The total emission rates of VOCs, total stored leaf and root terpenoid concentrations as well as metabolic fingerprints were calculated by summing the emission rates or concentrations of the individual compounds within each sample per metabolite group. The relative composition was calculated by dividing the emission rate or concentration of each compound by the corresponding total emission rate or total concentration for that sample. As measures of chemodiversity for VOCs, stored leaf and root terpenoid profiles, and leaf metabolic fingerprints, we calculated the richness and the functional Hill diversity (FHD, with *q* = 1). The richness was based on all compounds found in a sample, whereas the FHD was calculated based on annotated compounds only, as it integrates richness, evenness, and structural disparity (Petrén et al. 2023).

We compared the impact of treatment, chemotype, maternal origin, and their interactions on plant dry biomass, total concentrations, and richnesses of compound groups as well as all chemodiversity measures with linear permutation models. Models were built with each plant trait as the response variable, treatment, chemotype, maternal origin, and their interactions as predictors. To avoid the violation of normality and homoscedasticity, the significant effect of predictors was tested using permutation‐based ANOVA after removing multicollinearity on the most parsimonious models. Holm adjustment was implemented when more than two predictors remained in the models.

To study phenotypic plasticity in the strict sense, that is the environmentally contingent trait expression in a given maternal origin, we quantified the magnitude of the plastic response using the relative distance plasticity index (RDPI), ranging from 0 (no plasticity) to 1 (maximal plasticity), by RDPI = (|x_envc_—x_envs_|)/(|x_envc_ + x_envs_|), where X_envc_ and X_envs_ represent the different trait values of clones kept under control and stressed conditions, respectively (as previously done in Kleine et al. 2017, following Valladares et al. 2006). We analyzed the impact of treatment, chemotype, maternal origin as well as their interactions on RDPI of the different traits using permutated linear models with the same methods as used for the phenotypic and metabolic traits.

To visualize the absolute composition of features from each metabolite group, we used multivariate analyses on each dataset (VOCs, stored leaf and root terpenoids, leaf metabolic fingerprints) after normalization. To ensure comparability across each dataset, median normalization, cube‐root transformation, and Pareto scaling were applied. For the leaf metabolic fingerprint data, we extracted significant features (*p* < 0.05, FDR correction) with Tukey's tests performed in MetaboAnalyst (v5.0) (Pang et al. 2021). To assess treatment effects on the composition of each metabolite group, we performed Partial Least Squares Discriminant Analysis (PLS‐DA). When models could not be built (that is for stored leaf and root terpenoids), nonmetric multidimensional scaling (NMDS) using Bray–Curtis dissimilarity as a distance measure was instead performed based on the relative composition. On each distance matrix resulting from the NMDS, the differences among chemotypes and maternal origins were tested using Permutational Multivariate Analysis of Variance using distance matrices (PERMANOVA).

## 3 Results

### 3.1 Aboveground Biomass Reduced Under Single and Combined Stresses

**FIGURE 1:**
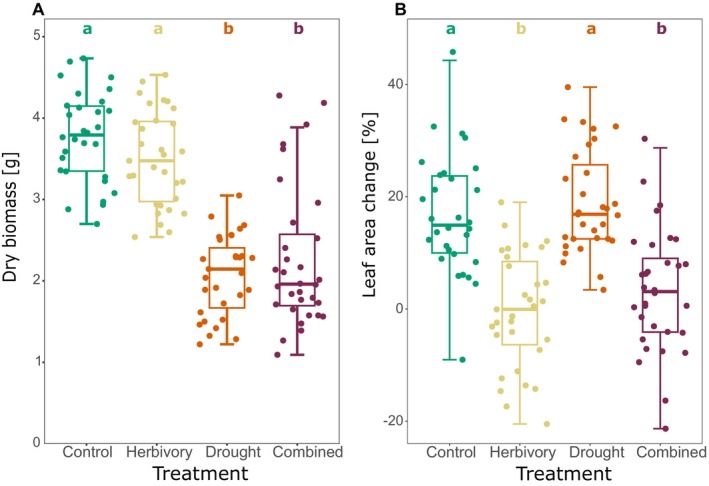
Dry biomass (A) and leaf area change (B; before and after herbivory treatment period) of *Tanacetum vulgare* plants exposed to different environmental stresses (combined: Drought and herbivory). Data presented as boxplots, with medians, interquartile ranges (IQR, boxes), and whiskers extending to the most extreme values with max. 1.5 times the IQR. Individual values are plotted as dots; *n* = 23 per treatment. Different letters indicate statistically significant differences (Tukey–Kramer *post hoc* test, adjusted *p* < 0.05 with Holm–Bonferroni method).

Aboveground dry biomass was significantly affected by stress treatment, with plants exposed to the drought and the combined stress treatment producing, on average, only one third of the biomass produced by control plants (Table 1, Figure 1A). Herbivory did not lead to a significant reduction in aboveground biomass. However, after application of herbivores, leaf area (in % of leaf area before application of caterpillars) did not increase in plants exposed to herbivory or combined stress, while it increased by 20% in control and drought‐stressed plants over the next 8 days (Figure 1B). About 20% of the caterpillars had developed into final‐instar larvae and did not feed anymore until the harvest of the plants. The aboveground dry biomass was also significantly impacted by the chemotype and maternal origin (Table 1, Figure S3), though there were no significant interactions with treatment.

**TABLE 1:** Summary of *F* and *p* values from ANOVAs testing the effects of treatment, chemotype, and maternal origin on different traits of *Tanacetum vulgare* .

Trait	Treatment (df = 3)		Chemotype (df = 3)		Maternal origin (df = 5)	
	*F*	*P*	*F*	*P*	*F*	*P*
Biomass	69.70	< 0.001	4.79	0.004	6.96	< 0.001
Leaf area change	29.87	< 0.001	5.10	0.004	—	—
VOC emission rate	14.79	< 0.001	—	—	5.12	< 0.001
VOC richness	22.83	< 0.001	—	—	14.14	< 0.001
VOC FHD^a^	22.06	< 0.001	9.41	< 0.001	9.22	< 0.001
Leaf terpenoid conc.^b^	—	—	80.00	< 0.001	—	—
Leaf terpenoid richness	—	—	4.57	0.005	10.37	< 0.001
Leaf terpenoid FHD	—	—	34.85	< 0.001	—	—
Root terpenoid conc.	—	—	16.20	< 0.001	—	—
Root terpenoid richness	—	—	9.38	< 0.001	—	—
Root terpenoid FHD	—	—	—	—	8.85	< 0.001
Leaf metabolic fingerprint richness	—	—	3.11	0.03	5.00	< 0.001
Leaf metabolic fingerprint FHD	—	—	—	—	3.72	0.004

*Note:* The effects were determined using ANOVA based on linear model permutation tests. The significance of predictors was calculated on the most parsimonious models after detecting multicollinearity. Effects marked with a hyphen were omitted from the model as being inapplicable for the model. The interactions of treatment, chemotype and/or maternal origin were omitted from the model as being inapplicable for the most parsimonious model.

^a^ FHD—functional Hill diversity.

^b^ Leaf and root terpenoids are stored terpenoids, conc.‐concentration.

A similar pattern was observed in the phenotypic plasticity (RDPI) of aboveground biomass, with distinct responses to the different treatments (Tables 2 and S3). Compared to the control plants, the RDPI of the aboveground biomass was higher in plants exposed to drought and the combined stresses, but to a lesser extent due to herbivory. However, biomass did not differ between plants of different chemotypes (Table 2) and maternal origins (Table 3).

**TABLE 2:** Means (standard deviations) of phenotypic plasticity (measured as RDPI^a^) of traits of *Tanacetum vulgare* of different chemotypes^b^, exposed to different stresses (combined: Drought and herbivory).

Trait	Treatment	Keto (*n* = 6)	Bthu (*n* = 8)	Aacet (*n* = 3)	Myrox (*n* = 6)	
Biomass	Herbivory	0.071 (0.026)	0.057 (0.014)	0.124 (0.061)	0.098 (0.033)	Treatment:
	Drought	0.292 (0.045)	0.359 (0.028)	0.144 (0.056)	0.259 (0.031)	*F* = 28.92
	Combined	0.359 (0.026)	0.283 (0.055)	0.184 (0.057)	0.248 (0.056)	*p <* 0.001
VOC emission rate	Herbivory	0.386 (0.080)	0.340 (0.075)	0.191 (0.096)	0.319 (0.124)	Treatment:
	Drought	0.371 (0.080)	0.304 (0.051)	0.236 (0.075)	0.280 (0.109)	*F* = 3.02
	Combined	0.557 (0.117)	0.553 (0.093)	0.408 (0.201)	0.323 (0.135)	*p* = 0.056
VOC richness	Herbivory	0.090 (0.027)	0.079 (0.026)	0.127 (0.047)	0.183 (0.080)	
	Drought	0.128 (0.053)	0.097 (0.033)	0.165 (0.118)	0.048 (0.023)	
	Combined	0.089 (0.029)	0.178 (0.044)	0.160 (0.012)	0.176 (0.075)	
VOC functional Hill diversity (FHD)	Herbivory	0.169 (0.060)	0.197 (0.046)	0.336 (0.143)	0.347 (0.118)	Chemotype:
	Drought	0.322 (0.096)	0.164 (0.034)	0.512 (0.220)	0.220 (0.035)	*F* = 3.06
	Combined	0.206 (0.045)	0.264 (0.070)	0.372 (0.130)	0.250 (0.098)	*p* = 0.04
Stored leaf terpenoid concentration	Herbivory	0.216 (0.094)	0.288 (0.075)	0.437 (0.198)	0.462 (0.125)	Chemotype:
	Drought	0.241 (0.081)	0.437 (0.073)	0.327 (0.085)	0.419 (0.085)	*F* = 2.99
	Combined	0.294 (0.094)	0.183 (0.048)	0.204 (0.054)	0.556 (0.098)	*p* = 0.040
Stored leaf terpenoid richness	Herbivory	0.090 (0.020)	0.117 (0.022)	0.102 (0.026)	0.110 (0.026)	
	Drought	0.081 (0.042)	0.112 (0.023)	0.104 (0.048)	0.100 (0.033)	
	Combined	0.133 (0.051)	0.125 (0.023)	0.076 (0.017)	0.185 (0.062)	
Stored leaf terpenoid FHD	Herbivory	0.076 (0.020)	0.187 (0.044)	0.044 (0.020)	0.209 (0.063)	Chemotype:
	Drought	0.056 (0.020)	0.095 (0.029)	0.150 (0.092)	0.157 (0.070)	*F* = 3.62
	Combined	0.111 (0.043)	0.160 (0.038)	0.115 (0.082)	0.259 (0.078)	*p* = 0.020
Stored root terpenoid concentration	Herbivory	0.176 (0.055)	0.198 (0.061)	0.143 (0.055)	0.288 (0.049)	
	Drought	0.339 (0.101)	0.279 (0.064)	0.293 (0.116)	0.193 (0.068)	
	Combined	0.302 (0.105)	0.346 (0.085)	0.238 (0.062)	0.285 (0.080)	
Stored root terpenoid richness	Herbivory	0.044 (0.023)	0.063 (0.024)	0.061 (0.043)	0.099 (0.018)	
	Drought	0.077 (0.028)	0.110 (0.014)	0.041 (0.006)	0.064 (0.016)	
	Combined	0.070 (0.011)	0.059 (0.007)	0.133 (0.067)	0.039 (0.011)	
Stored root terpenoid FHD	Herbivory	0.058 (0.029)	0.128 (0.049)	0.173 (0.079)	0.054 (0.034)	
	Drought	0.103 (0.021)	0.070 (0.020)	0.138 (0.022)	0.038 (0.013)	
	Combined	0.246 (0.105)	0.056 (0.022)	0.236 (0.109)	0.125 (0.088)	
Leaf metabolic fingerprint richness	Herbivory	0.030 (0.011)	0.027 (0.009)	0.034 (0.016)	0.013 (0.007)	Chemotype:
	Drought	0.030 (0.012)	0.023 (0.007)	0.045 (0.020)	0.013 (0.004)	*F* = 4.74
	Combined	0.026 (0.010)	0.019 (0.006)	0.056 (0.020)	0.012 (0.002)	*p* = 0.007
Leaf metabolic fingerprint FHD	Herbivory	0.133 (0.057)	0.145 (0.051)	0.137 (0.065)	0.075 (0.022)	
	Drought	0.163 (0.049)	0.134 (0.042)	0.122 (0.044)	0.099 (0.017)	
	Combined	0.093 (0.044)	0.123 (0.026)	0.183 (0.085)	0.050 (0.021)	

^a^ RDPI: (|x_envc_ – x_envs_|)/(|x_envc_ + x_envs_|) where X_envc_ and X_envs_ represent the different trait values of clones kept under control and stressed conditions.

^b^ Chemotypes dominated by artemisia ketone (Keto), β‐thujone (Bthu), artemisyl acetate, artemisia ketone and artemisia alcohol (Aacet) or (*Z*)‐myroxide, santolina triene and artemisyl acetate (Myrox). Data are evaluated for each of the four chemotypes, with the numbers in parentheses representing the number of replicates for each. Significant effects of predictors are based on ANOVA results of the most parsimonious models (only significant effects are shown).

**TABLE 3:** Mean (standard deviation) of phenotypic plasticity (measured as RDPI) of *Tanacetum vulgare* traits from six maternal origins, exposed to different stresses (combined: Drought and herbivory).

Trait	Treatment	16 (*n* = 4)	18 (*n* = 6)	23 (*n* = 4)	26 (*n* = 3)	7 (*n* = 3)	8 (*n* = 3)	
Dry biomass	Herbivory	0.098 (0.060)	0.044 (0.009)	0.061 (0.029)	0.094 (0.035)	0.073 (0.027)	0.104 (0.043)	Treatment:
	Drought	0.232 (0.082)	0.342 (0.060)	0.258 (0.070)	0.254 (0.031)	0.349 (0.040)	0.311 (0.086)	*F* = 28.92
	Combined	0.282 (0.087)	0.206 (0.082)	0.244 (0.087)	0.290 (0.059)	0.356 (0.034)	0.286 (0.072)	*p <* 0.001
VOC emission rate	Herbivory	0.120 (0.049)	0.308 (0.061)	0.343 (0.099)	0.328 (0.088)	0.235 (0.145)	0.585 (0.141)	Treatment:
	Drought	0.428 (0.176)	0.263 (0.120)	0.210 (0.049)	0.267 (0.064)	0.296 (0.086)	0.378 (0.099)	*F* = 3.02
	Combined	0.335 (0.196)	0.550 (0.134)	0.419 (0.137)	0.415 (0.109)	0.464 (0.167)	0.584 (0.264)	*p* = 0.056
VOC richness	Herbivory	0.173 (0.100)	0.089 (0.045)	0.143 (0.044)	0.055 (0.023)	0.055 (0.024)	0.251 (0.117)	
	Drought	0.077 (0.007)	0.075 (0.038)	0.059 (0.024)	0.081 (0.045)	0.214 (0.082)	0.097 (0.097)	
	Combined	0.192 (0.079)	0.270 (0.053)	0.107 (0.045)	0.056 (0.009)	0.176 (0.041)	0.213 (0.136)	
VOC functional Hill diversity (FHD)	Herbivory	0.285 (0.153)	0.135 (0.047)	0.249 (0.117)	0.180 (0.064)	0.230 (0.077)	0.230 (0.083)	
	Drought	0.361 (0.066)	0.100 (0.075)	0.177 (0.061)	0.241 (0.073)	0.332 (0.173)	0.321 (0.089)	
	Combined	0.251 (0.079)	0.266 (0.132)	0.222 (0.120)	0.138 (0.054)	0.233 (0.080)	0.187 (0.029)	
Stored leaf terpenoid concentration	Herbivory	0.274 (0.245)	0.205 (0.081)	0.526 (0.097)	0.422 (0.115)	0.302 (0.115)	0.135 (0.105)	
	Drought	0.441 (0.078)	0.262 (0.107)	0.424 (0.136)	0.282 (0.090)	0.440 (0.102)	0.396 (0.121)	
	Combined	0.422 (0.213)	0.229 (0.037)	0.213 (0.097)	0.474 (0.117)	0.165 (0.036)	0.286 (0.106)	
Stored leaf terpenoid richness	Herbivory	0.122 (0.011)	0.079 (0.016)	0.134 (0.040)	0.103 (0.027)	0.118 (0.022)	0.073 (0.037)	Maternal origin:
	Drought	0.181 (0.019)	0.183 (0.067)	0.101 (0.025)	0.073 (0.025)	0.082 (0.025)	0.012 (0.012)	*F* = 3.07
	Combined	0.214 (0.094)	0.175 (0.081)	0.126 (0.036)	0.149 (0.048)	0.117 (0.037)	0.032 (0.016)	*p* = 0.020
Stored leaf terpenoid FHD	Herbivory	0.102 (0.087)	0.053 (0.026)	0.164 (0.040)	0.197 (0.059)	0.214 (0.082)	0.060 (0.022)	
	Drought	0.114 (0.095)	0.139 (0.049)	0.125 (0.071)	0.110 (0.067)	0.109 (0.037)	0.043 (0.018)	
	Combined	0.289 (0.065)	0.185 (0.047)	0.153 (0.080)	0.177 (0.083)	0.115 (0.046)	0.097 (0.052)	
Stored root terpenoid concentration	Herbivory	0.238 (0.091)	0.095 (0.044)	0.209 (0.119)	0.233 (0.058)	0.231 (0.032)	0.215 (0.097)	
	Drought	0.208 (0.125)	0.379 (0.048)	0.346 (0.066)	0.263 (0.100)	0.112 (0.069)	0.377 (0.132)	
	Combined	0.302 (0.072)	0.081 (0.033)	0.261 (0.124)	0.231 (0.066)	0.488 (0.060)	0.491 (0.161)	
Stored root terpenoid richness	Herbivory	0.125 (0.024)	0.039 (0.022)	0.100 (0.035)	0.072 (0.018)	0.017 (0.017)	0.051 (0.051)	
	Drought	0.060 (0.015)	0.103 (0.026)	0.087 (0.028)	0.066 (0.030)	0.097 (0.018)	0.074 (0.025)	
	Combined	0.011 (0.011)	0.040 (0.002)	0.095 (0.030)	0.057 (0.011)	0.102 (0.040)	0.081 (0.010)	
Stored root terpenoid FHD	Herbivory	0.040 (0.021)	0.031 (0.013)	0.214 (0.092)	0.076 (0.043)	0.142 (0.043)	0.043 (0.014)	
	Drought	0.065 (0.021)	0.084 (0.037)	0.090 (0.033)	0.065 (0.026)	0.070 (0.038)	0.114 (0.020)	
	Combined	0.060 (0.037)	0.119 (0.067)	0.167 (0.083)	0.164 (0.119)	0.090 (0.069)	0.276 (0.142)	
Leaf metabolic fingerprint richness	Herbivory	0.005 (0.003)	0.029 (0.007)	0.032 (0.017)	0.026 (0.012)	0.035 (0.012)	0.019 (0.013)	
	Drought	0.014 (0.005)	0.019 (0.010)	0.025 (0.015)	0.024 (0.013)	0.040 (0.013)	0.023 (0.008)	
	Combined	0.017 (0.003)	0.023 (0.012)	0.021 (0.012)	0.022 (0.010)	0.038 (0.020)	0.018 (0.009)	
Leaf metabolic fingerprint FHD	Herbivory	0.029 (0.020)	0.093 (0.022)	0.163 (0.072)	0.133 (0.055)	0.192 (0.076)	0.080 (0.043)	
	Drought	0.107 (0.034)	0.096 (0.089)	0.115 (0.031)	0.183 (0.040)	0.132 (0.069)	0.108 (0.033)	
	Combined	0.064 (0.046)	0.098 (0.037)	0.108 (0.054)	0.080 (0.044)	0.207 (0.034)	0.054 (0.037)	

*Note:* Data are evaluated for each of the six maternal origins. Numbers in the header row indicate the different maternal origins, with the numbers in parentheses representing the number of replicates for each. Significant effects of predictors are based on ANOVA results of the most parsimonious models (only significant effects are shown).

### 3.2 VOCs Impacted by Single and Combined Stresses

**FIGURE 2:**
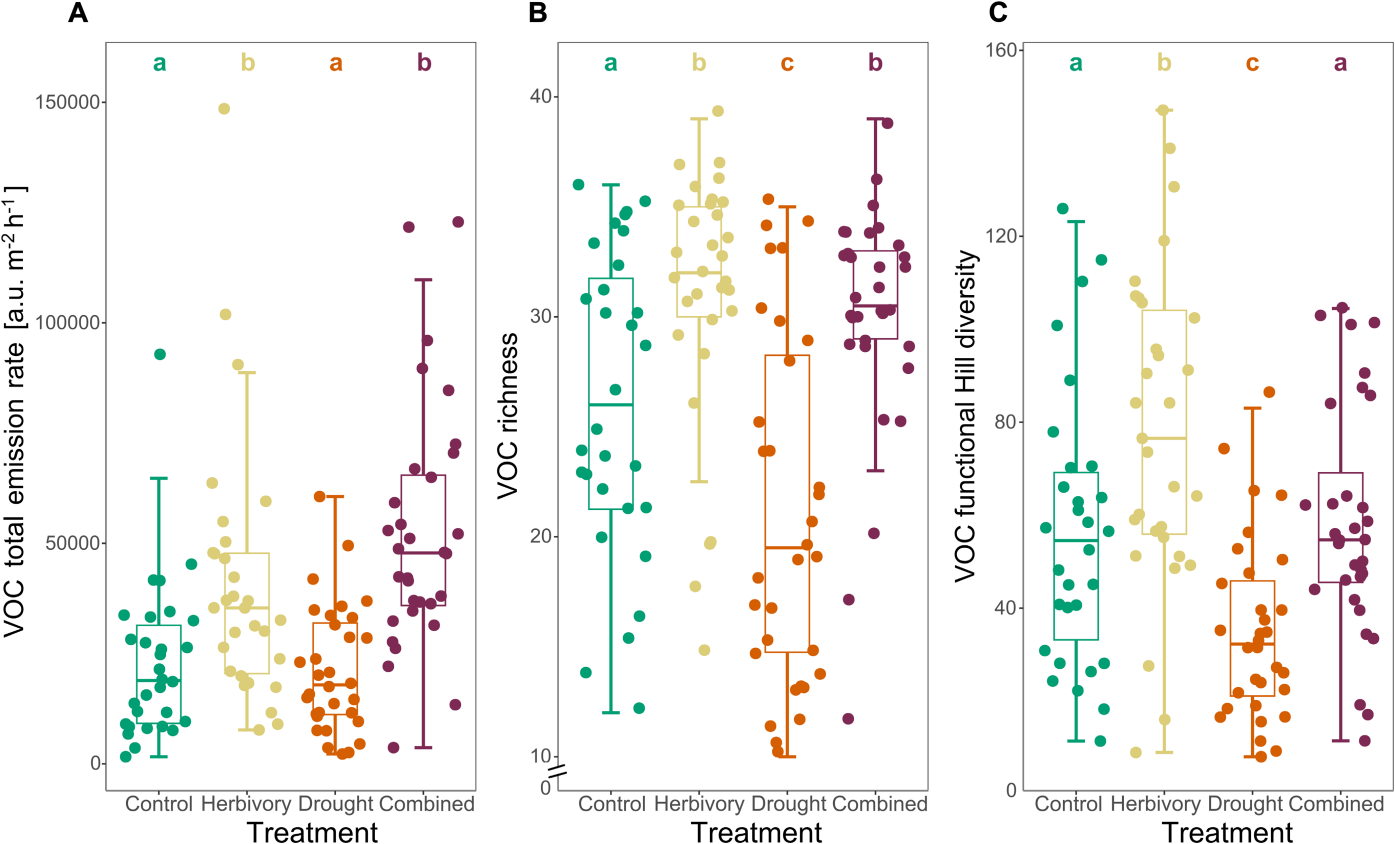
Volatile organic compounds (VOCs) emitted from *Tanacetum vulgare* plants exposed to different environmental stresses (combined: Drought and herbivory). Data for total emission rate (A; a.u. – arbitrary units, normalized), richness (B), and functional Hill diversity (C) are presented as boxplots, with medians, interquartile ranges (IQR, boxes), and whiskers extending to the most extreme values with max. 1.5 times the IQR. Individual values are plotted; *n* = 23 per treatment. Different letters indicate statistically significant differences (Tukey–Kramer *post hoc* test, adjusted *p* < 0.05 with Holm–Bonferroni method).

A total of 54 VOCs were detected in all samples (a detailed list of all compounds can be found in the DataPlant file with all data for this study). Total emission rate, richness, and FHD of VOCs were all significantly affected by treatment (Table 1). The emission rate was approximately twice as high in plants exposed to herbivory or the combined treatment than in plants of the other two treatment groups (Figure 2A). Compared with control plants, the VOC richness was higher in plants exposed to herbivory or combined stresses and lower in plants subjected to drought (Figure 2B). No VOC was exclusively released by plants of one of the treatments, but some VOCs, such as methyl salicylate and the sesquiterpenoids α‐copaene, β‐copaene, and germacrene D, were less frequently emitted by drought‐stressed plants, while these same VOCs were more frequently released upon herbivory alone or in combination with drought. Herbivory and drought stress had contrasting effects on VOC FHD. Herbivory led to a significant increase, whereas drought stress resulted in a decrease in FHD. However, the VOC FHD of the combined stress treatment did not differ significantly from the control (Figure 2C). Furthermore, chemotype and maternal origin significantly affected VOC emission rate, richness, and FHD (Table 1). Total emission rates were higher in the Keto chemotype than in the Bthu chemotype (Figure S4A). Plants of the Bthu and Myrox chemotypes had a higher VOC FHD than those of the Keto chemotype (Figure S4E). Plants of maternal origin 8 had lower VOC emission rates than those of maternal origin 18 (Figure S4B). Similarly, plants of maternal origin 8 exhibited lower VOC richness and FHD than those of maternal origins 16 and 18 (Figure S4D,F). However, interactions between treatment and either chemotype or maternal origin were not significant. Thus, VOC responses to stress were consistent across chemotypes and maternal origins.

The RDPI of the total VOC emission rate differed marginally between the various treatments (Table 2). It was influenced by biotic and abiotic stress, while higher values were noticed under the combined stresses compared to the single stress treatments. In contrast, the RDPI of the VOC emission rate was not affected by chemotype or maternal origin. Only the RDPI of the VOC FHD was affected by chemotype, whereas VOC richness was neither affected by treatment, chemotype, or maternal origin (Tables 2 and 3).

**FIGURE 3:**
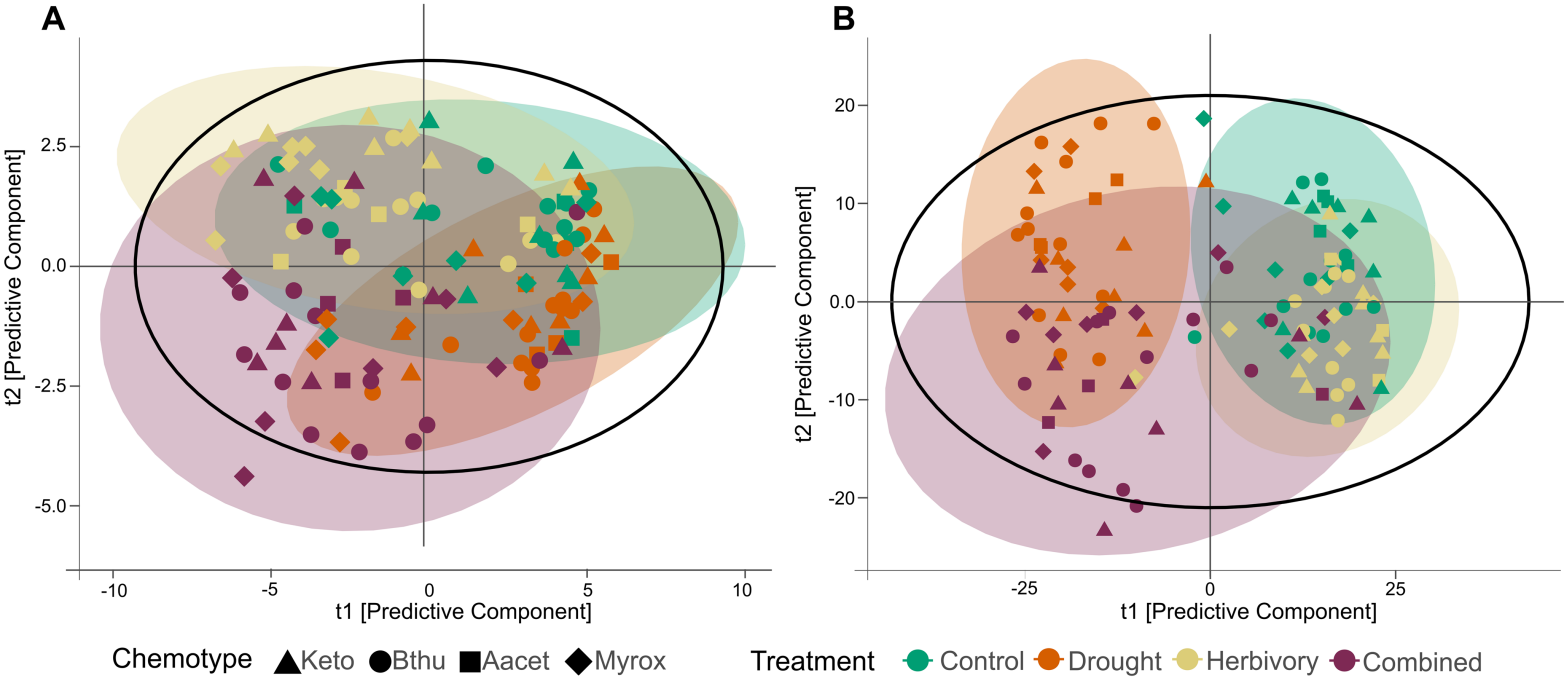
Partial least squares‐discriminant analysis (PLSD‐DA) plot of the composition of VOCs (A; R^2^X = 0.35, R^2^Y = 0.28, Q^2^ = 0.18) and leaf metabolic fingerprints (B; R^2^X = 0.34, R^2^Y = 0.38, Q^2^ = 0.32) of *Tanacetum vulgare* plants exposed to different environmental stresses (combined: Drought and herbivory; *n* = 23 per treatment). Chemotypes dominated by artemisia ketone (Keto, mono chemotype; *n* = 24), β‐thujone (Bthu, mono chemotype; *n* = 32), artemisyl acetate, artemisia ketone and artemisia alcohol (Aacet, mixed chemotype; *n* = 12) or (*Z*)‐myroxide, santolina triene and artemisyl acetate (Myrox, mixed chemotype; *n* = 24).

A PLS‐DA of the VOC emission profiles revealed a separation particularly between drought‐stressed plants and those of the control or herbivory treatment groups (Figure 3A). The VOC composition of plants exposed to the combined stresses overlapped with the VOC profiles of the plants exposed to the individual stresses. The score plot accounted for 35% of total variance and had a predictive ability of *Q* ^2^ = 0.182.

### 3.3 Stored Leaf and Root Terpenoids Unaffected by Treatments

A total of 23 and 20 stored terpenoids were detected by GC–MS in the leaves and roots, respectively (for details see DataPlant file). Leaf profiles were dominated by monoterpenoids, with only a few sesquiterpenoids, whereas root profiles mostly comprized sesquiterpenoids. Treatment had no effect on the concentration, richness, or FHD of leaf and root terpenoids (Table 1). As expected, leaf stored terpenoid profiles differed significantly in terms of concentration, richness, and FHD between chemotypes, while their richness was also influenced by maternal origin (Table 1). Plants of the Keto chemotype showed the highest concentration of stored leaf terpenoids, whereas those of the Myrox chemotype showed the lowest concentration (Figure S5A). No significant differences in richness were observed across chemotypes based on pairwise comparisons (Figure S5C), although chemotype had a significant effect in the most parsimonious model (Table 1). Plants of the Aacet chemotype displayed a lower FHD of stored leaf terpenoids than those of the mono chemotypes Keto and Bthu, while the mixed Myrox chemotype exhibited the highest FHD (Figure S5E). Plants of maternal origins 7 and 8 displayed a lower stored terpenoid richness than those of maternal origins 18 and 26 (Figure S5D).

Root‐stored terpenoids differed in concentration and richness among chemotypes, while their FHD was only affected by maternal origin (Table 1). Specifically, the mixed chemotype Myrox showed higher terpenoid concentrations and richness than other chemotypes (Figure S6A,C). The FHD of root‐stored terpenoids was higher in plants of maternal origin 8 than in those of most other origins, except origin 23 (Figure S6F).

In terms of RDPI, treatments had no influence on the concentration, richness, and FHD of either leaf or root terpenoids. The RDPI of the concentration and FHD of leaf terpenoids differed among chemotypes (Table 2), whereas the RDPI of concentration, richness, and FHD of root terpenoids was not affected by treatment, chemotype, or maternal origin (Table 2).

**FIGURE 4:**
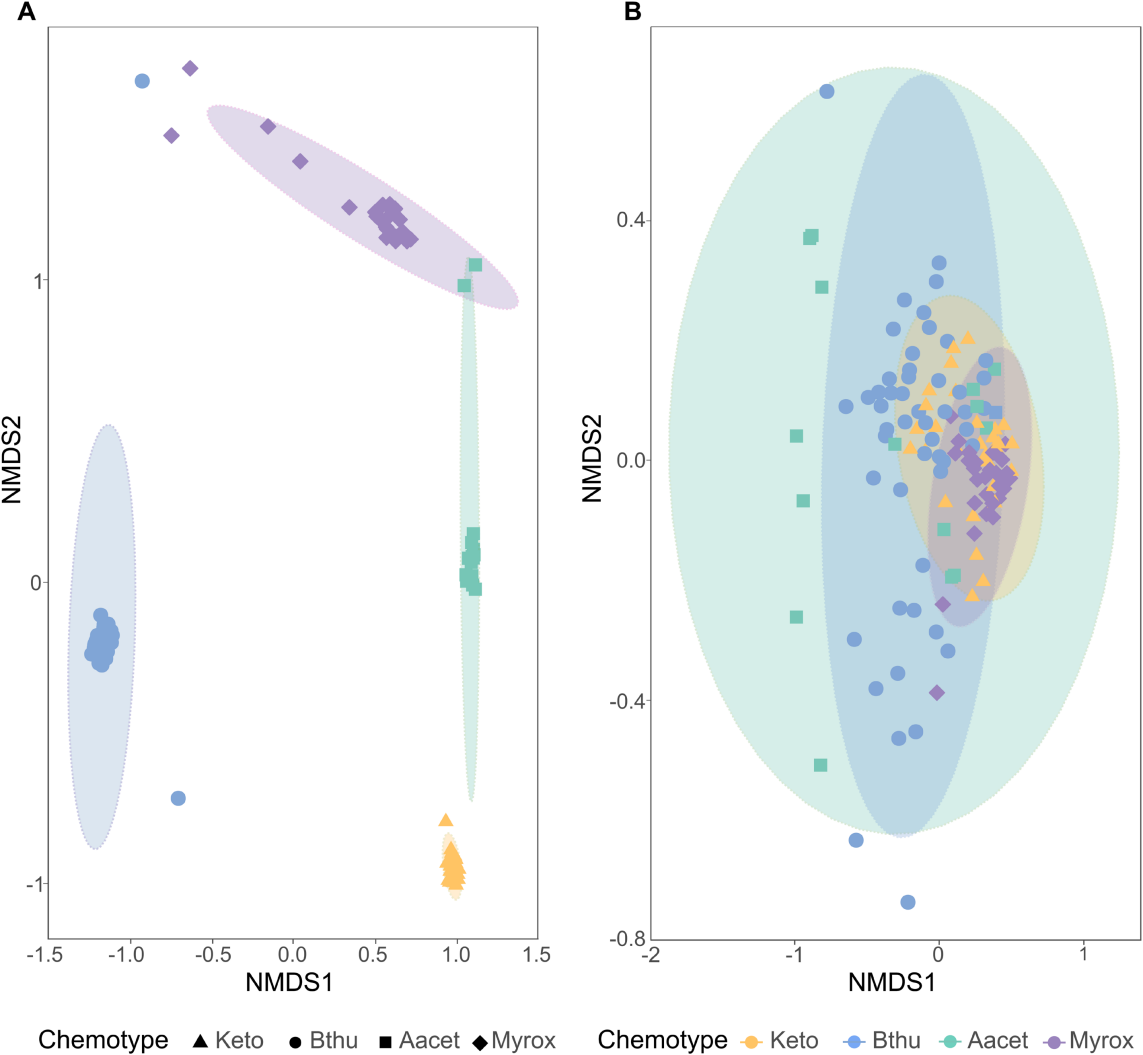
Nonmetric multidimensional scaling (NMDS) plot of the composition of stored terpenoids of leaves (A) and roots (B) of *Tanacetum vulgare* plants across chemotypes. Chemotypes dominated by artemisia ketone (Keto, mono chemotype, *n* = 24), β‐thujone (Bthu, mono chemotype, *n* = 32), artemisyl acetate, artemisia ketone and artemisia alcohol (Aacet, mixed chemotype, *n* = 12) or (*Z*)‐myroxide, santolina triene and artemisyl acetate (Myrox, mixed chemotype, *n* = 24).

The leaf terpenoid composition differed among chemotypes, while the root terpenoid composition showed a less pronounced separation between chemotypes, as depicted in an NMDS (Figure 4).

### 3.4 Leaf Metabolic Fingerprints Modulated by Treatments

Untargeted LC–MS analysis, representing leaf metabolic fingerprints, yielded 2927 mass features (for details see DataPlant file). Of these, 1102 mass features differed significantly in abundance among treatments (Tukey's test, *p* < 0.05, FDR correction). However, we found no variation in the richness or diversity of metabolic fingerprints across treatments (Table 1). Instead of treatment effects, chemotype, and maternal origin influenced the richness of metabolic fingerprints, while FHD was affected only by maternal origin. Plants of the mixed chemotype Myrox had a higher leaf metabolic richness than those of the Keto chemotype (Figure S7A). Plants of maternal origin 18 showed a higher metabolic fingerprint richness than those of maternal origins 7, 16, and 26 (Figure S7B), as well as a higher FHD than those of maternal origin 23 (Figure S7D).

Treatment had no effect on the RDPI of the leaf metabolic fingerprint (Table 2), but the RDPI of the fingerprint richness was impacted by the chemotype.

The composition of the leaf metabolic fingerprints could clearly be separated by treatment (Table S4), as shown in the PLS‐DA score plot (Figure 3B). Drought‐stressed plants formed a distinct group, fully separated from control and herbivory‐treated plants, while plants of the combined stress treatment showed overlaps with the other groups. However, leaf metabolic fingerprints could not be grouped according to chemotype or maternal origin (Table S4).

## 4 Discussion

Increasing climate variability, particularly the increased frequency and severity of drought events (Gebrechorkos et al. 2025), is changing the environmental conditions in which plants grow, defend themselves, and interact with herbivores. This study demonstrates that although drought restricted growth and changed the metabolic composition in *T. vulgare* , herbivory substantially enhanced VOC emissions. These responses occurred largely independently of chemotype and maternal origin, suggesting that predominantly environmental conditions shape plastic plant traits. Baseline chemodiversity was tied to genetic background, implying that both plastic and constitutive chemical traits may impact ecological responses to global climate change.

### 4.1 Drought Limits Growth but Does Not Constrain Plasticity

The drought period had a significant impact on the production of plant biomass in drought‐ and combined‐stressed plants, with *T. vulgare* plants growing only by about one third compared to those not exposed to drought. Drought is well known to suppress aboveground biomass production, potentially due to limited photosynthesis caused by stomata closing to prevent water loss or by direct damage to the photosynthetic machinery (Chaves et al. 2003; Sharma and Dubey 2005; Okamoto et al. 2013). A significantly reduced biomass allocation to aboveground tissues has been observed across numerous species, while the investment in roots usually increases in response to drought (Eziz et al. 2017). This distinct allocation can lead to a higher root‐to‐shoot ratio, as previously found in *T. vulgare* in response to drought (Kleine and Müller 2014). In comparison to the impact of drought on biomass, the biomass loss due to herbivory for 5 days was negligible in our plants. Larvae of *S. exigua* are highly polyphagous (Merkx‐Jacques et al. 2008), but only led to a somewhat reduced gain of leaf area but no significant biomass reduction in the herbivory‐ and combined stress‐treated plants. Thus, in contrast to our hypothesis, the combined stresses did not result in a more pronounced reduction in biomass than the drought stress alone. Strikingly, RDPI values were, on average, highest in plants exposed to drought or the combined stress, indicating that drought does not constrain plasticity.

### 4.2 Herbivory Drives VOC Induction, While Drought Modulates Chemodiversity

Nevertheless, herbivory was sufficient to lead to a measurable increase in total VOC emission rates compared to plants without herbivory. In response to herbivory, numerous plant species have been shown to enhance their VOC emissions (Gols 2014), which can act as a direct defense against herbivores or an indirect defense by attracting predators (Abbas et al. 2022). However, little is known about VOC emissions when plants are additionally subjected to other abiotic stresses, such as drought (but see Lin et al. 2023). Under such combined stresses, the VOC emission rates of *T. vulgare* plants were similarly high as in plants impacted only by herbivory, indicating that the induction of VOC biosynthesis was mainly driven by herbivory. In contrast, a more pronounced enhancement of VOC emissions in response to combined drought and herbivory, and thus a synergistic effect, has been observed in *Alnus glutinosa* trees (Copolovici et al. 2014) and herbs such as *Solanum lycopersicum* (Lin et al. 2022). Nevertheless, plant responses to such combined stresses also depend on the duration of herbivory (Lin et al. 2022) and the severity of drought, probably due to distinct priming by different drought intensities and thus specific hormonal crosstalk when plants are exposed to both drought and (simulated) herbivory (Scott et al. 2019).

Plants that were only exposed to drought exhibited a reduced VOC richness and diversity compared to control *T. vulgare* . Similar reductions in VOC richness and diversity due to drought have been observed in several grassland species, while some species showed an increase, indicating highly species‐specific responses (Reinecke et al. 2024). Under drought, plants may redirect their biosynthetic machinery towards other metabolites that offer better protection against water limitation (Reinecke et al. 2024). In nature, drought stress is often related to heat stress. Heat stress alone has been found to result in enhanced emissions of monoterpenes, likely originating from damage to permanent storage structures (Nagalingam et al. 2023). Since we found decreases rather than increases in VOC richness and no changes in VOC emission rates in response to drought, we assume this treatment did not damage the storage structures. VOC richness increased significantly due to herbivory and the combined treatment in our experiment, indicating that VOC profiles did not only change in quantity, but that some metabolites were more frequently produced upon herbivory, such as α‐copaene and methyl salicylate, which are known to directly repel herbivores or attract natural enemies (James 2005; Magnani et al. 2025). Combined stresses led, on average, to the highest RDPI values in VOC emission rates, highlighting that VOC emission is a very plastic trait. Other VOCs, such as α‐longipinene, γ‐terpinene, artemisia alcohol, and (*E*)‐4,8‐dimethylnona‐1,3,7‐triene, were emitted from herbivory‐ and combined‐stressed plants in higher rates than from controls. These VOCs can act as defences against several herbivores and microorganisms (Estell et al. 2004; Ivănescu et al. 2021), but are also known to attract some herbivore species (Clavijo McCormick et al. 2016; Zhao et al. 2024).

Furthermore, a higher VOC FHD was observed in *T. vulgare* plants subjected to herbivory compared to those of the other treatments. Higher chemodiversity of metabolites can benefit plants, for example, by attracting more pollinators or reducing herbivore pressure (Salazar et al. 2016; Ziaja and Müller 2023; Sasidharan et al. 2024). Overall, the VOC composition across plants from different chemotypes and maternal origins showed consistent responses to the treatments, with some chemotype‐ or maternal origin‐specific effects, but with no interaction with treatment. This suggests that the VOC induction upon herbivory is highly conserved in *T. vulgare* .

### 4.3 Stored Terpenoids Show Low Responsiveness

Unlike VOCs, stored leaf and root terpenoids of *T. vulgare* were not significantly affected by treatment. Defence reallocation may only occur over time or under more pronounced herbivory and may depend on leaf age, in line with assumptions of the optimal defence hypothesis. Such allocation patterns have been, for example, observed in cotton (Eisenring et al. 2017). A previous study on *T. vulgare* found that a 12‐day drought exposure led to higher stored terpenoid concentrations in leaves but reduced concentrations in roots, compared to well‐watered plants. Meanwhile, 8 days of herbivory by *Mamestra brassicae* did not affect leaf terpenoids but led to increased root terpenoid concentrations (Kleine and Müller 2014). Simulating a systematic acquired resistance response in *T. vulgare* using pipecolic acid, which mimics the action of biotrophic pathogens, also enhanced the concentrations of root stored sesquiterpenoids (Rahimova et al. 2025), demonstrating that biotic stress can result in an induction of root terpenoid biosynthesis. In our current study, a shorter duration and less pronounced herbivory than in the previous studies may have prevented the root‐stored terpenoids from responding to herbivory. In contrast, we here applied drought for a longer period and more severely, which may have hindered the transient responses of root‐stored terpenoids. Previous work on *S. lycocarpum* also revealed a fine‐tuned responsiveness of root terpenoids to leaf herbivory, which depended on the drought severity (Mundim et al. 2021). In *Pseudotsuga menziesii* , root terpenoid concentrations increased with moderate drought but not with severe drought. Responsiveness also differed between provenances with distinct climates, indicating potential roles of terpenoids in stress resistance in this species (Kleiber et al. 2017).

### 4.4 Metabolic Reprogramming and Ecological Implications

While stored leaf terpenoids did not change due to treatment in *T. vulgare* , they, of course, pronouncedly differed between chemotypes, since chemotypes were assigned according to the leaf terpenoid composition. This was reflected in differences in stored terpenoid concentration, richness, and FHD. Plants of the mixed Myrox chemotype showed the highest FHD of stored leaf terpenoids, as previously also found when these chemotypes were grown in the field (Ziaja and Müller 2025). Thus, the high FHD of leaf terpenoids in this chemotype appears to be relatively consistent under varying environmental conditions. Furthermore, plants of the Myrox chemotype exhibited comparably high RDPI values for leaf terpenoid concentrations. This observation is in line with the hypothesis by Petrén et al. (2024) that predicts that plants with higher chemodiversity exhibit a higher plasticity in adjusting their chemical profiles in response to environmental changes, enabling flexible defence strategies. Similarly, root terpenoid concentrations differed in terms of concentration, richness, and FHD across chemotypes, although chemotype was not included in the most parsimonious model for FHD. Our results underscore the importance of considering intraspecific chemical variation when assessing plant responses to stress factors.

The leaf metabolic fingerprints of *T. vulgare* are not primarily determined by the terpenoid chemotypes, but rather by the maternal origin (Dussarrat et al. 2023). Consistent with this, maternal origin significantly impacted leaf metabolic richness and FHD in the present study. Nevertheless, with regard to the richness of metabolic fingerprints, plastic responses differed across chemotypes, indicating that intraspecific variation in responsiveness occurs in a trait‐specific manner. While metabolite biosynthesis is often regulated by environmental conditions, it is primarily determined by the underlying genetics (Zhan et al. 2022); in *T. vulgare* , it is reflected by chemotype and maternal origin. Terpenoid chemotypes are also present in other species, such as *Solidago gigantea* , *Thymus vulgaris* , or *Quercus suber* (Johnson et al. 2007; Loreto et al. 2009; Linhart et al.,2005). Interestingly, the chemotypes or genotypes of a wide range of species exhibit distinct responses to drought or herbivory (Calf et al. 2019; de Simón et al. 2017; Karban, Orrock, et al. 2016), which may indicate adaptive differentiation (Castells and Sanchez‐Martinez 2025). This highlights the fascinating intra‐ and interspecific variation in phenotypic plasticity in response to environmental challenges.

## 5 Conclusion

The biomass and the overall leaf metabolic composition revealed clearly distinct responses to drought, herbivory, and their combination in *T. vulgare* . Changes in metabolic fingerprints in response to different stresses and stress combinations have only been studied in a few species (Kutyniok and Müller 2012; Sun et al. 2015; Tiziani et al. 2022). Ideally, different species should be exposed to the same stresses in order to identify general versus species‐specific response patterns in plant traits. Such studies should be conducted in a controlled environment simulating realistic natural scenarios to ensure comparability and reproducibility (Vanzo et al. 2015). Notably, the contrasting responses of VOCs, stored terpenoids, and metabolic fingerprints in *T. vulgare* emphasize the importance of examining specific metabolite classes when assessing plant responses. Moreover, phenotypic plasticity can vary considerably due to chemotype and maternal origin, or genotype. Our study contributes to a deeper understanding of the mechanisms underlying plant adaptation, especially under scenarios of climate change.

## Author Contributions

X.X. analysis of data, draft of result section; T.D. plant harvest, analysis of LC‐MS data; L.B. preparation of samples for chemical analyses; D.Z. and T.D. terpenoid analyses; Y.B.S. and B.W. VOC analysis; R.J. plant preparation; J.B.W. support in conceptualization, performance of experiment, phenotypic data analysis; J.‐P.S. support in conceptualization, performance of experiment, VOC analysis; C.M. funding acquisition, conceptualization, set‐up of experiment, manuscript writing, draft of introduction, methods and discussion section. All authors contributed to editing the manuscript.

## Supporting information


**Table S1:** *Tanacetum vulgare* plant individuals chosen for the experiment.
**Table S2:** Watering regime for plants of the different treatment groups.
**Table S3:** Means (standard deviations of *n* = 23) of phenotypic plasticity (measured as RDPI) of traits of *Tanacetum vulgare* , exposed to different stresses (combined: drought and herbivory).
**Table S4:** Results of permutational multivariate analysis of variance (PERMANOVA) testing the effects of treatment, chemotype, and maternal origin on the leaf metabolic fingerprints of *Tanacetum vulgare* .
**Figure S1:** Timeline for experiment.
**Figure S2:** Distribution of plants in climate chambers.
**Figure S3:** Dry biomass of *Tanacetum vulgare* plants of different chemotypes (A) and maternal origins (B).
**Figure S4:** Total emission rate, richness, and functional Hill diversity of volatile organic compounds of *Tanacetum vulgare* in dependence of chemotype and maternal origin.
**Figure S5:** Concentration, richness, and functional Hill diversity of stored leaf terpenoids of *Tanacetum vulgare* in dependence of chemotype and maternal origin.
**Figure S6:** Concentration, richness, and functional Hill diversity of stored root terpenoids of *Tanacetum vulgare* in dependence of chemotype and maternal origin.
**Figure S7:** Concentration, richness, and functional Hill diversity of leaf metabolic fingerprints of *Tanacetum vulgare* in dependence of chemotype and maternal origin.

## Data Availability

The data are available via DataPLANT: https://git.nfdi4plants.org/xue.xiao/DPPN_SICO_project/‐/tree/df5b6d8b724b75f0a8e19d7a928ea26f23ee336a/.
