# Supplementary material for: Plastic Responses to Single and Combined Environmental Stresses in a Highly Chemodiverse Aromatic Plant Species
Source: Physiol Plant. 2025 Nov 5;177(6):e70626. doi: 10.1111/ppl.70626 (PMC12588736; doi:10.1111/ppl.70626)
Supplement: Supplementary file 1 — Table S1: Tanacetum vulgare plant individuals chosen for the experiment. Table S2: Watering regime for plants of the different treatment groups. Table S3: Means (standard deviations of n = 23) of phenotypic plasticity (measured as RDPI) of traits of Tanacetum vulgare , exposed to different stresses (combined: drought and herbivory). Table S4: Results of permutational multivariate analysis of variance (PERMANOVA) testing the effects of treatment, chemotype, and maternal origin on the leaf metabolic fingerprints of Tanacetum vulgare . Figure S1: Timeline for experiment. Figure S2: Distribution of plants in climate chambers. Figure S3: Dry biomass of Tanacetum vulgare plants of different chemotypes (A) and maternal origins (B). Figure S4: Total emission rate, richness, and functional Hill diversity of volatile organic compounds of Tanacetum vulgare in dependence of chemotype and maternal origin. Figure S5: Concentration, richness, and functional Hill diversity of stored leaf terpenoids of Tanacetum vulgare in dependence of chemotype and maternal origin. Figure S6: Concentration, richness, and functional Hill diversity of stored root terpenoids of Tanacetum vulgare in dependence of chemotype and maternal origin. Figure S7: Concentration, richness, and functional Hill diversity of leaf metabolic fingerprints of Tanacetum vulgare in dependence of chemotype and maternal origin. [file PPL-177-e70626-s001.pdf]

Supplementary Material

Plastic responses to single and combined environmental stresses in a highly chemodiverse aromatic plant species

Xue Xiao[1], Thomas Dussarrat[1], Dominik Ziaja[1], Yonca B. Seymen[2], Lukas Brokate[1], Ruth Jakobs[1], Baris Weber[2], Jana Barbro Winkler[2], Jörg-Peter Schnitzler[2], Caroline Müller[1,3,*]

[1]Bielefeld University, Universitätsstr. 25, 33615 Bielefeld

[2]Research Unit Environmental Simulation, Helmholtz Zentrum Munich, 85764 Neuherberg, Germany

[3] Joint Institute for Individualisation in a Changing Environment (JICE), University of Münster and Bielefeld University, Bielefeld, Germany

* Corresponding author: caroline.mueller@uni-bielefeld.de

Table S1: *Tanacetum vulgare* plant individuals chosen for the experiment.

Maternal genotype	Chemotype			
	Keto	BThu	Aacet	Myrox
7			7_22 (1)	7_33 (2)
8	8_42 (2)	8_32 (3)		
16		16_2 (3)	16_40 (1)	
		16_23 (4)		
18	18_5 (4)			18_46 (4)
23		23_6 (3)	23_42 (2)	
26	26_26 (2)			26_165 (1)

Plant individuals belonged to six maternal genotypes and four foliar chemotypes. The chemotypes were dominated (> 50% of total terpenoid composition) by artemisia ketone (Keto), β-thujone (Bthu), artemisyl acetate, artemisia ketone and artemisia alcohol (Aacet) or (*Z*)-myroxide, santolina triene and artemisyl acetate (Myrox). Numbers in parentheses indicate the number of treatment replicates.

Table S2: Watering regime for plants of the different treatment groups from onset of drought.

Day	Treatment groups	
	Control and herbivory stress	Drought and combined stress
0 (start)	75 x 2 (suppliers per pot) = 150 mL	0
2	90 x 2 = 180 mL	90 x 2 = 180 mL
4	90 x 2 = 180 mL	0
6	90 x 2 = 180 mL	45 x 2 = 90 mL
8	90 x 2 = 180 mL	45 x 2 = 90 mL
10	100 x 2 =200 mL	50 x 2 =100 mL
12	200 mL	100 mL
14	200 mL	0 mL
15	0 mL	100 mL
16	200 mL	100 mL
19	200 mL	100 mL

Control and herbivory-stressed plants received the same amount of water (1870 mL) and drought-stressed and drought- and herbivory-stressed plants experienced the same watering regime (860 mL). Soil moisture and plant status were regularly monitored to assure drought stress. Two days prior to the start of the drought stress treatment 150 ml water with 0.25% Hakaphos Rot fertiliser was applied per plant.

Table S3: Means (standard deviations of n = 23) of phenotypic plasticity (measured as RDPI) of traits of *Tanacetum vulgare*, exposed to different stresses (combined: drought and herbivory).

**Trait**	**Herbivory**	**Drought**	**Combined**	
Dry biomass	0.080 (0.014)	0.287 (0.023)	0.281 (0.027)	**Treatment:** $F = 28.92p < 0.001$
VOC emission rate	0.318 (0.047)	0.297 (0.037)	0.453 (0.060)	**Treatment:** $F = 3.02$, $p = 0.056$
VOC richness	0.116 (0.025)	0.101 (0.023)	0.152 (0.026)	
VOC functional Hill diversity (FHD)	0.215 (0.034)	0.254 (0.041)	0.207 (0.032)	
Stored leaf terpenoid conc.	0.334 (0.055)	0.367 (0.042)	0.312 (0.049)	
Stored leaf terpenoid richness	0.106 (0.012)	0.100 (0.016)	0.136 (0.022)	
Stored leaf terpenoid FHD	0.145 (0.026)	0.108 (0.024)	0.167 (0.029)	
Stored root terpenoid conc.	0.209 (0.030)	0.274 (0.040)	0.304 (0.044)	
Stored root terpenoid richness	0.067 (0.012)	0.080 (0.010)	0.066 (0.010)	
Stored root terpenoid FHD	0.096 (0.024)	0.079 (0.012)	0.147 (0.040)	
Leaf metabolic fingerprint richness	0.025 (0.005)	0.025 (0.005)	0.024 (0.005)	
Leaf metabolic fingerprint FHD	0.123 (0.024)	0.131 (0.020)	0.104 (0.020)	

conc. - concentration

Table S4: Results of permutational multivariate analysis of variance (PERMANOVA) testing the effects of treatment, chemotype, and maternal origin on the leaf metabolic fingerprints of *Tanacetum vulgare*.

	**F**	**R^2**	***p***	***p. adj***
Treatment	5.58	0.13	0.004	0.009
Chemotype	2.36	0.06	0.080	0.100
Maternal origin	1.83	0.07	0.110	0.110

[1]PERMANOVA used Bray-Curtis distance metrics.

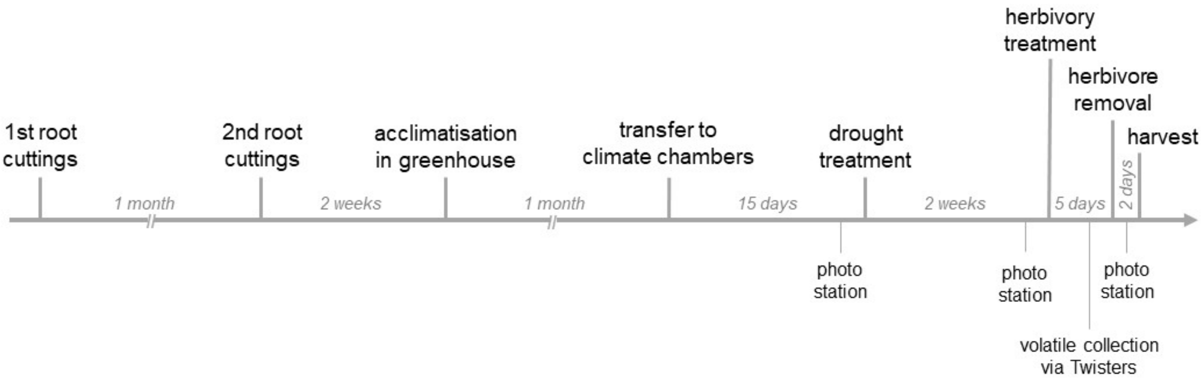

Figure S1: Timeline for experiment. The timeline shows which tasks were performed during certain time intervals.

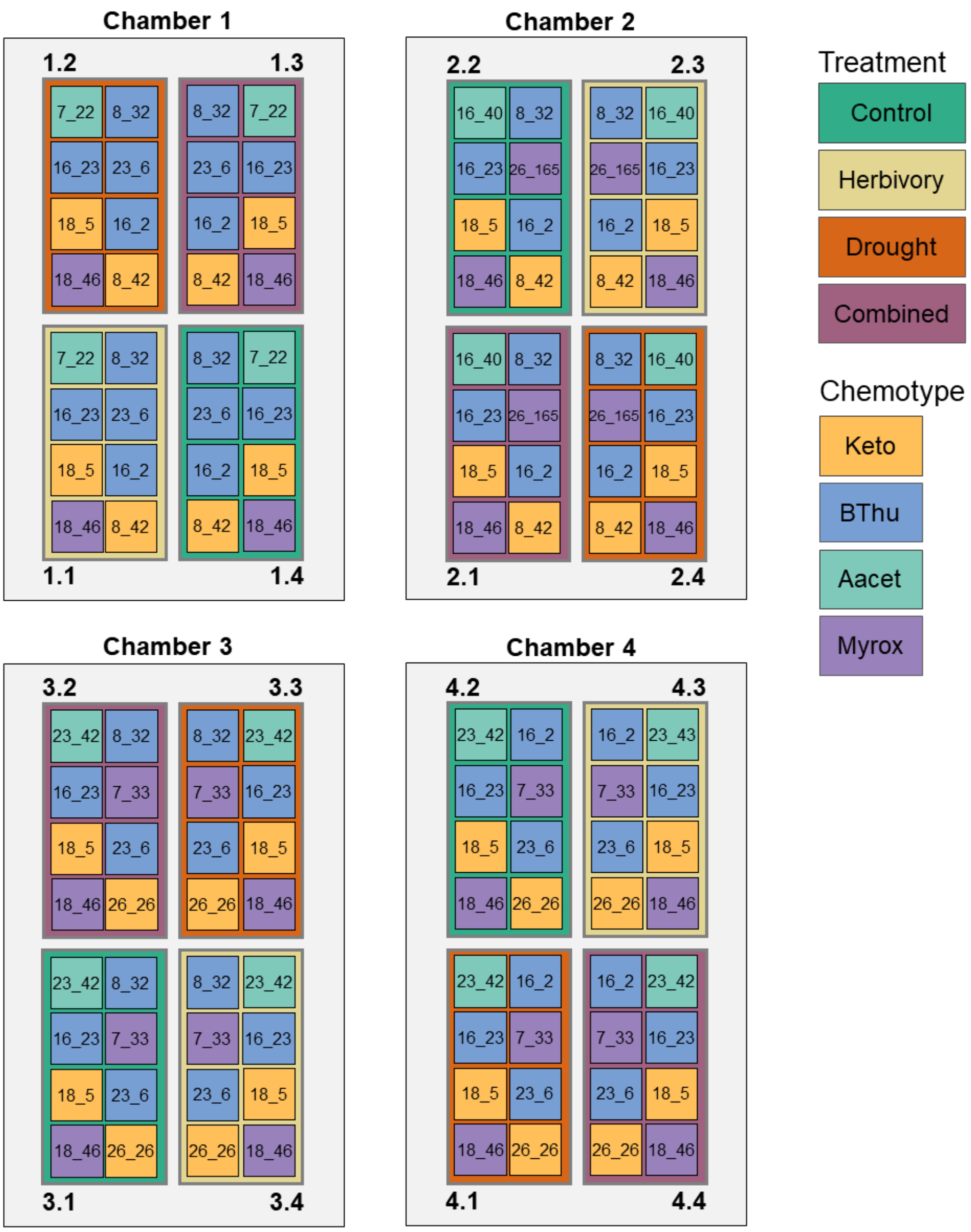

Figure S2. Set-up of plants in experimental chambers. The experimental set-up consisted of four chambers, with four sub-chambers in each chamber. Clonal plants experienced one of the four treatments control, drought, insect herbivory, and combined stresses (drought and herbivory) in one of four sub-chambers within the main chambers. Each clone had one plant ID, as indicated as numbers in the boxes, and belonged to a specific chemotype (indicated by colour of box) and maternal origin (first number of plant ID).

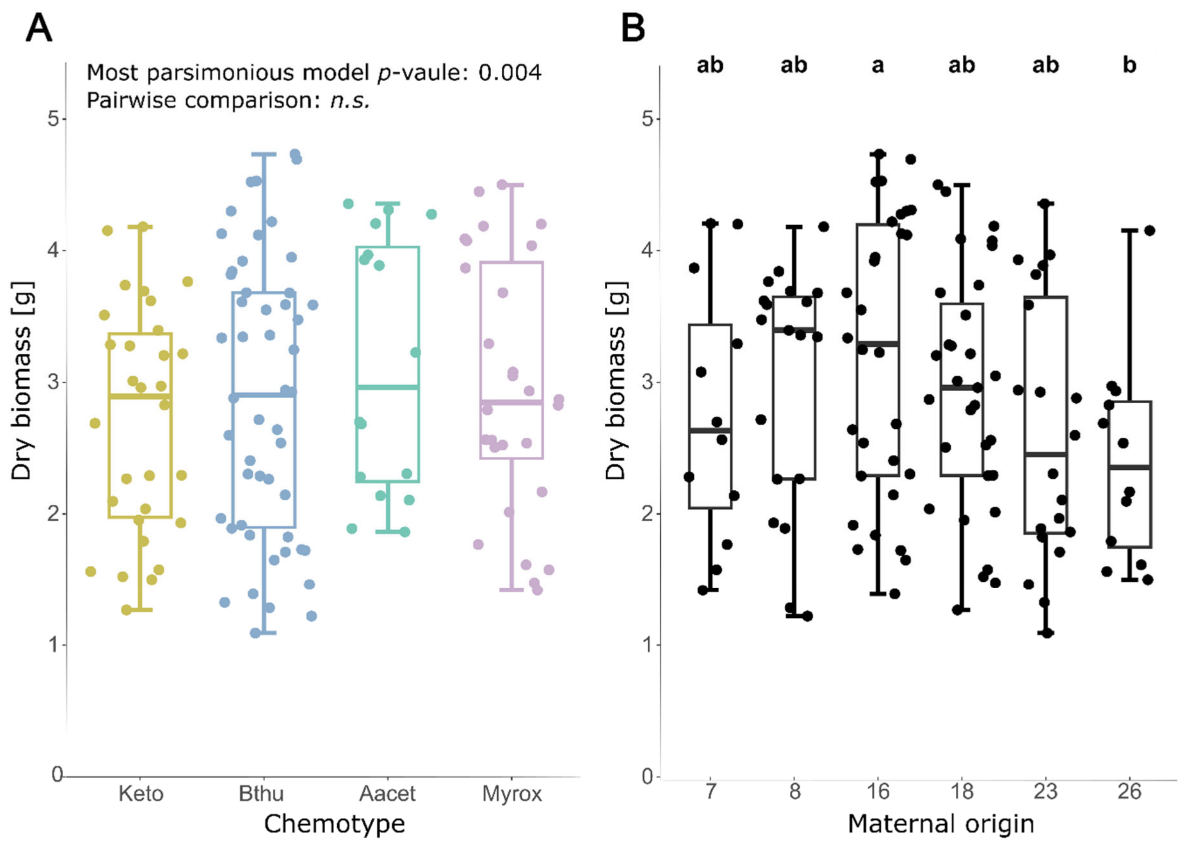

Figure S3: Dry biomass of *Tanacetum vulgare* plants of different chemotypes (A) and maternal origins (B) at the final harvest. Chemotypes dominated by artemisia ketone (Keto), β-thujone (Bthu), artemisyl acetate, artemisia ketone and artemisia alcohol (Aacet) or (*Z*)-myroxide, santolina triene and artemisyl acetate (Myrox). Data presented as boxplots, with medians, interquartile ranges (IQR, boxes), and whiskers extending to the most extreme values with max. 1.5 times the IQR. Individual values are plotted as points. Different letters indicate statistically significant differences (Tukey-Kramer *post hoc* test, adjusted $p < 0.05$ with Holm–Bonferroni method).

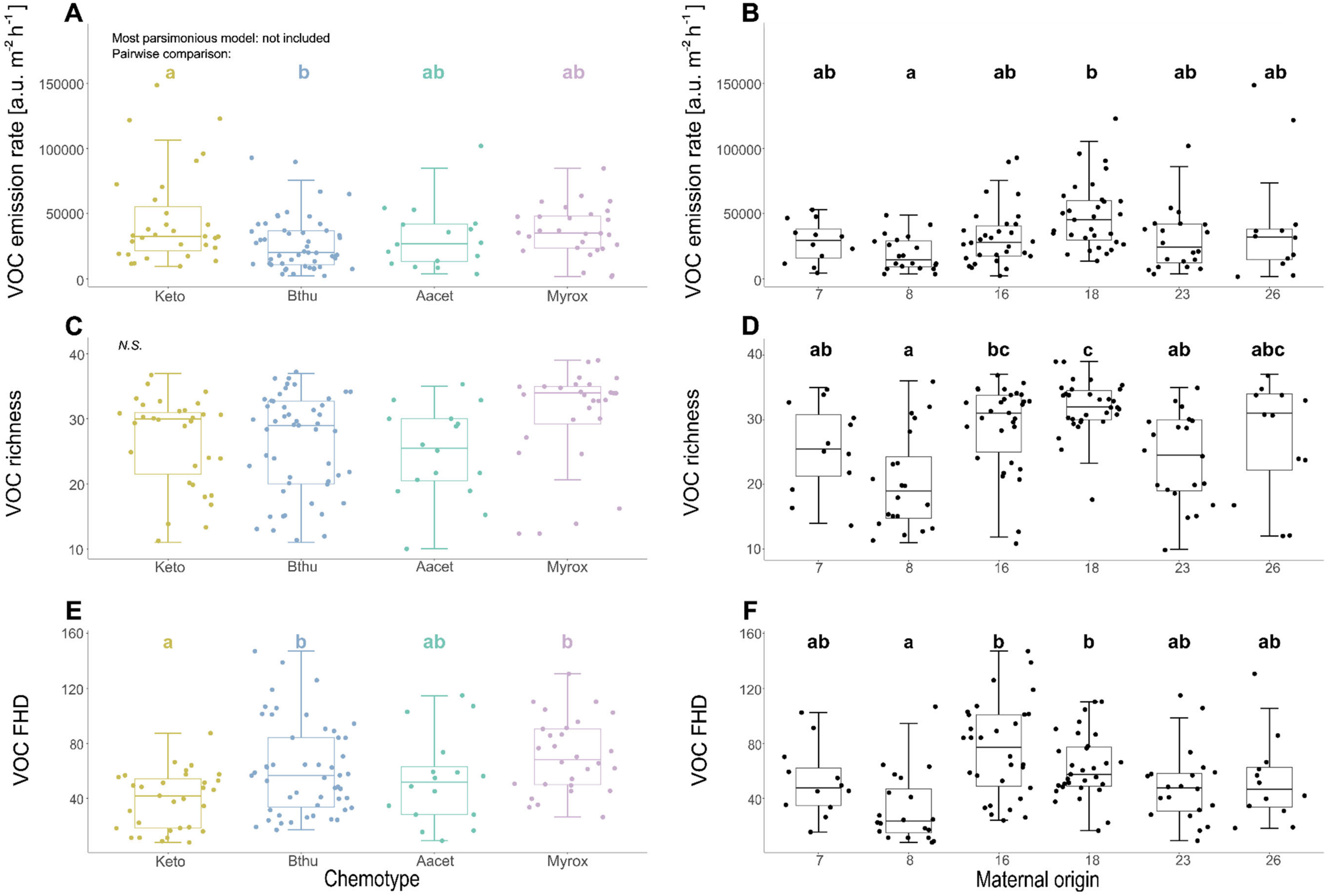
A
Most parsimonious model: not included
Pairwise comparison:
a
b
ab
ab
VOC emission rate [a.u. m^{-2} h^{-1}]
150000
100000
50000
0
Keto
Bthu
Aacet
Myrox
B
ab
a
ab
b
ab
ab
VOC emission rate [a.u. m^{-2} h^{-1}]
150000
100000
50000
0
7
8
16
18
23
26
C
N.S.
VOC richness
40
30
20
10
Keto
Bthu
Aacet
Myrox
D
ab
a
bc
c
ab
abc
VOC richness
40
30
20
10
7
8
16
18
23
26
E
a
b
ab
b
VOC FHD
160
120
80
40
Keto
Bthu
Aacet
Myrox
Chemotype
F
ab
a
b
b
ab
ab
VOC FHD
160
120
80
40
7
8
16
18
23
26
Maternal origin

63

Figure S4: Emission rates (A, B), richness (C, D) and functional Hill diversity (FHD) (E, F) of volatile organic compounds (VOCs) of *Tanacetum vulgare* plants of different chemotypes (A, C, E) and maternal origins (B, D, F). Chemotypes dominated by artemisia ketone (Keto), β-thujone (Bthu), artemisyl acetate, artemisia ketone and artemisia alcohol (Aacet) or (*Z*)-myroxide, santolina triene and artemisyl acetate (Myrox). Data presented as boxplots, with medians, interquartile ranges (IQR, boxes), and whiskers extending to the most extreme values with max. 1.5 times the IQR. Individual values are plotted as points. Different letters indicate statistically significant differences (Tukey-Kramer *post hoc* test, adjusted $p < 0.05$ with Holm–Bonferroni method).

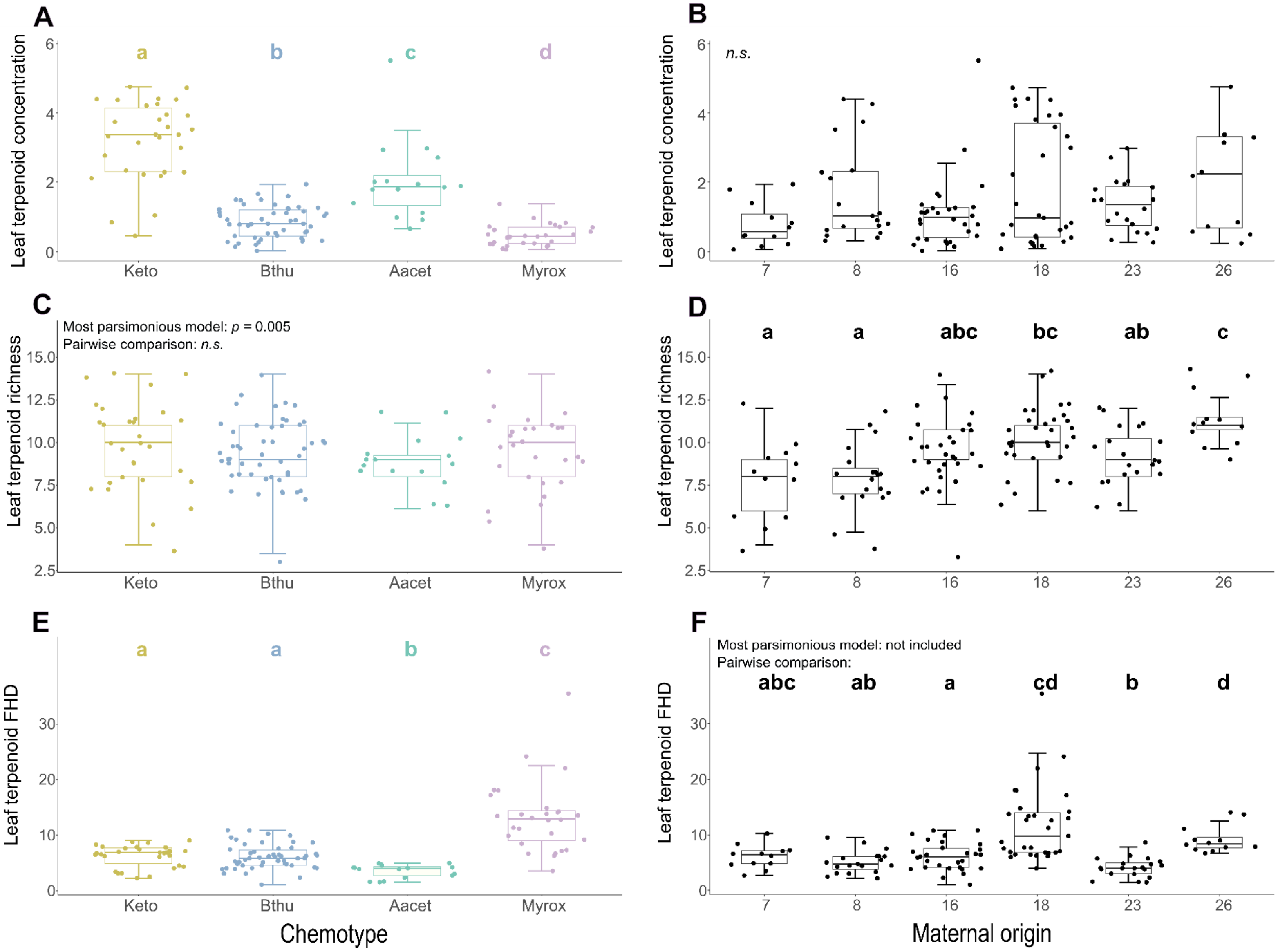
A
Leaf terpenoid concentration
a b c d
Keto Bthu Aacet Myrox
B
n.s.
7 8 16 18 23 26
C
Most parsimonious model: p = 0.005
Pairwise comparison: n.s.
Leaf terpenoid richness
D
a a abc bc ab c
E
Leaf terpenoid FHD
a a b c
Chemotype
F
Most parsimonious model: not included
Pairwise comparison:
abc ab a cd b d
Maternal origin

70

Figure S5: Concentration (A, B), richness (C, D) and functional Hill diversity (FHD) (E, F) of stored leaf terpenoids of *Tanacetum vulgare* plants of different chemotypes (A, C, E) and maternal origins (B, D, F). Chemotypes dominated by artemisia ketone (Keto), β-thujone (Bthu), artemisyl acetate, artemisia ketone and artemisia alcohol (Aacet) or (*Z*)-myroxide, santolina triene and artemisyl acetate (Myrox). Data presented as boxplots, with medians, interquartile ranges (IQR, boxes), and whiskers extending to the most extreme values with max. 1.5 times the IQR. Individual values are plotted as points. Different letters indicate statistically significant differences (Tukey-Kramer *post hoc* test, adjusted $p < 0.05$ with Holm–Bonferroni method).

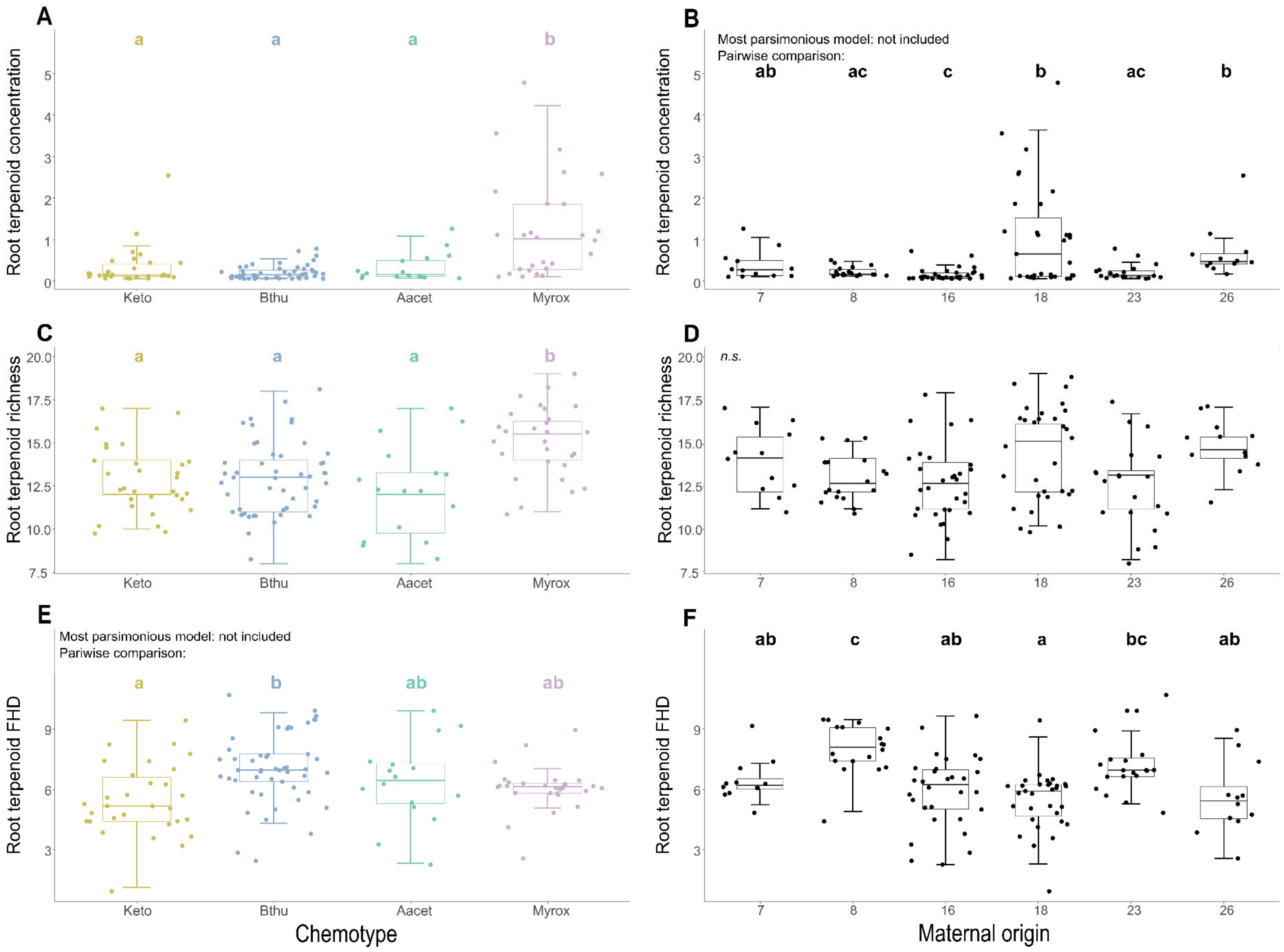
A
B
C
D
E
F
Most parsimonious model: not included
Pairwise comparison:
n.s.
Most parsimonious model: not included
Pariwise comparison:
Root terpenoid concentration
Root terpenoid richness
Root terpenoid FHD
Keto
Bthu
Aacet
Myrox
Chemotype
Maternal origin

Figure S6: Concentration (A, B), richness (C, D) and functional Hill diversity (FHD) (E, F) of stored root terpenoids of *Tanacetum vulgare* plants of different chemotypes (A, C, E) and maternal origins (B, D, F). Chemotypes dominated by artemisia ketone (Keto), β-thujone (Bthu), artemisyl acetate, artemisia ketone and artemisia alcohol (Aacet) or (*Z*)-myroxide, santolina triene and artemisyl acetate (Myrox). Data presented as boxplots, with medians, interquartile ranges (IQR, boxes), and whiskers extending to the most extreme values with max. 1.5 times the IQR. Individual values are plotted as points. Different letters indicate statistically significant differences (Tukey-Kramer *post hoc* test, adjusted p < 0.05 with Holm–Bonferroni method).

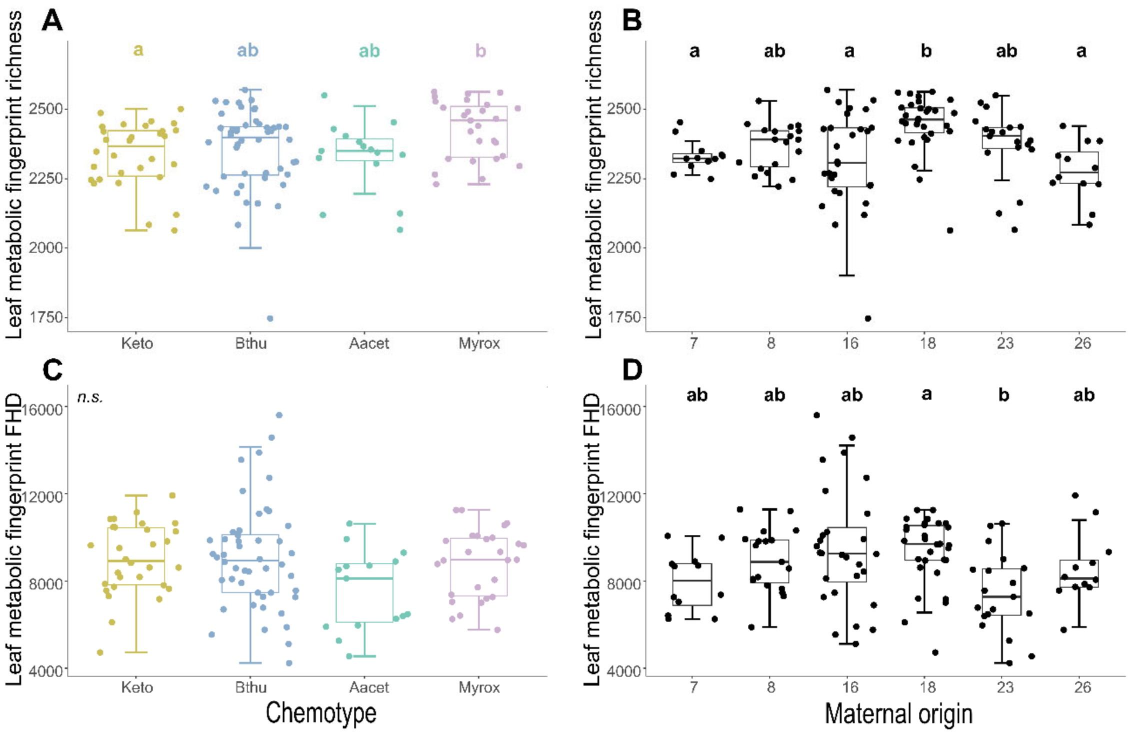

Figure S7: Richness (A, B) and functional Hill diversity (C, D) of leaf metabolic fingerprints of *Tanacetum vulgare* plants of different chemotypes (A, C) and maternal origins (B, D). Chemotypes dominated by artemisia ketone (Keto), β-thujone (Bthu), artemisyl acetate, artemisia ketone and artemisia alcohol (Aacet) or (*Z*)-myroxide, santolina triene and artemisyl acetate (Myrox). Data presented as boxplots, with medians, interquartile ranges (IQR, boxes), and whiskers extending to the most extreme values with max. 1.5 times the IQR. Individual values are plotted as points. Different letters indicate statistically significant differences (Tukey-Kramer *post hoc* test, adjusted $p < 0.05$ with Holm–Bonferroni method).
